# Evidences for a Nutritional Role of Iodine in Plants

**DOI:** 10.3389/fpls.2021.616868

**Published:** 2021-02-17

**Authors:** Claudia Kiferle, Marco Martinelli, Anna Maria Salzano, Silvia Gonzali, Sara Beltrami, Piero Antonio Salvadori, Katja Hora, Harmen Tjalling Holwerda, Andrea Scaloni, Pierdomenico Perata

**Affiliations:** ^1^Plant Lab, Institute of Life Sciences, Scuola Superiore Sant’Anna, Pisa, Italy; ^2^Proteomics and Mass Spectrometry Laboratory, Institute for the Animal Production System in the Mediterranean Environment (ISPAAM), National Research Council, Napoli, Italy; ^3^Institute of Clinical Physiology, National Research Council, Pisa, Italy; ^4^SQM International N.V., Antwerpen, Belgium

**Keywords:** iodine, Arabidopsis, plant growth, plant nutrition, plant phenotype, transcriptomics, proteomics

## Abstract

Little is known about the role of iodine in plant physiology. We evaluated the impact of low concentrations of iodine on the phenotype, transcriptome and proteome of *Arabidopsis thaliana*. Our experiments showed that removal of iodine from the nutrition solution compromises plant growth, and restoring it in micromolar concentrations is beneficial for biomass accumulation and leads to early flowering. In addition, iodine treatments specifically regulate the expression of several genes, mostly involved in the plant defence response, suggesting that iodine may protect against both biotic and abiotic stress. Finally, we demonstrated iodine organification in proteins. Our bioinformatic analysis of proteomic data revealed that iodinated proteins identified in the shoots are mainly associated with the chloroplast and are functionally involved in photosynthetic processes, whereas those in the roots mostly belong and/or are related to the action of various peroxidases. These results suggest the functional involvement of iodine in plant nutrition.

## Introduction

Plants need macro- and micro-nutrients for their growth and development. Nutrients are chemical elements that are components of biological molecules and/or influence essential metabolic functions. The elements that to date are considered as plant nutrients are C, H, O, N, P, K (primary nutrients), Ca, Mg, S (secondary nutrients), and Fe, Zn, Cu, Mn, B, Cl, Mo, Co, and Ni (micro-nutrients) (Mengel and Kirkby, 2001).

Halogens are the least represented chemical group of plant micro-nutrients, chloride being the only micro-nutrient currently recognised in plant physiology, due to its regulatory action in proton-transfer reactions in the photosystem II (Brahmachari et al., 2018). Studying the effect of different concentrations and forms of iodine on the growth of several crops of agricultural importance, Borst Pauwels (1961) referred to iodine as a micro-nutrient for plant, and a similar conclusion was derived by Lehr et al. (1958) working on tomato.

A growing number of recent studies reporting the effect of iodine on plant growth have focused on the benefit of increasing iodine content in plants as a biofortificant in human and animal health (Medrano-Macías et al., 2016; Gonzali et al., 2017). Plant tissues generally increase their iodine content following its exogenous administration. However, the presence of iodine as a trace element/contaminant in the soil/nutrient solution/atmosphere cannot be avoided, thus preventing the effects related to the presence/absence of this element from being easily observed (Fuge and Johnson, 1986; Ashworth, 2009). The functional role of iodine as a plant nutrient might therefore have been masked.

Plants can absorb iodine from roots or above ground structures (stomata and cuticular waxes) (Medrano-Macías et al., 2016; Gonzali et al., 2017), translocate it mainly through the xylematic route and volatilise it as methyl iodide (CH_3_I) through the action of halide ion-methyltransferase (HMT) and halide/thiol methyltransferase (HTMT) enzymes (Medrano-Macías et al., 2016; Gonzali et al., 2017).

Little is known about the chemical forms of iodine inside plant tissues. Inorganic iodine, in particular iodide (I^–^), however, seems to be predominant (Weng et al., 2008). Plants can also incorporate iodine into organic molecules, such as iodosalicylates, iodobenzoates (Smoleń et al., 2020), monoiodotyrosine (MIT), di-iodotyrosine (DIT) and triiodothyronine (T3) (Eales, 1997; Smoleń et al., 2020). Interestingly, MIT and DIT have a key role in the physiology of vertebrates, as they are precursors of the two thyroid hormones (THs) triiodothyronine (T3) and L-thyroxine (T_4_) as part of the thyroglobulin protein (Zimmermann et al., 2008).

In plants, the presence of a thyroglobulin-like protein has never been reported, and the metabolic role of MIT, DIT and T3 molecules, if any, and their biosynthetic mechanism are still unknown. Nevertheless, protein iodination has been verified in several seaweed species (Hou et al., 2000; Romarís-Hortas et al., 2014), even if it has not yet been demonstrated in plants.

Iodine is likely involved in several physiological and biochemical processes (Medrano-Macías et al., 2016; Gonzali et al., 2017). The presence of low concentrations of iodine is often associated with beneficial effects on plant growth, production and stress resistance, whereas toxic effects are observed when applying iodine at high concentrations, especially in the I^–^ form, which is more phytotoxic than iodate (IO_3_^–^) (Voogt et al., 2010; Medrano-Macías et al., 2016; Gonzali et al., 2017; Incrocci et al., 2019). Thresholds for beneficial or toxic concentrations have been reported for all micro-nutrients (Welch and Shuman, 1995). Interestingly, the concentrations of iodine added to nutrient solutions that have been associated with positive effects for plants (ranging from approximately 10^2^–10^4^ nM) (Medrano-Macías et al., 2016; Gonzali et al., 2017) are comparable with those generally effective for other elements described as plant micro-nutrients (Sonneveld, 2002), suggesting that iodine may play a similar role in plant nutrition.

We explored the role of iodine as a nutrient for plants using various experimental approaches. Our results showed that iodine, when supplied at a well-defined concentration range, positively affected the phenotype of *Arabidopsis thaliana* plants, and altered the organism’s transcriptome. Most importantly, protein iodination was observed for the first time. These results are strongly suggestive of the role of iodine as a plant nutrient.

## Materials and Methods

### Plant Material and Cultivation System

Plants of *Arabidopsis thaliana*, ecotype Columbia 0, *Solanum lycopersicum* L., cv. Micro-Tom, *Lactuca sativa* L., var. crispa, *Triticum turgidum* L., var. durum, and *Zea mays* L., var. saccharata, were used in the experiments, as summarised in Supplementary Figure S1.

The cultivation protocol commonly applied in all the experiments is described as follows: seeds of the different species were sown on rockwool plugs and vernalised for 3 days. After this period, plants were hydroponically cultivated in a growth chamber (22°C day/18°C night with a 12 h photoperiod, a quantum irradiance of 100 μmol photons m^–2^ s^–1^ and a relative humidity close to 35%), in a floating system. A base nutrient solution, renewed once a week, was prepared minimising iodine contamination by dissolving in MilliQ water the following amounts of ultrapure salts: 1.25 mM KNO_3_, 1.50 mM Ca(NO_3_)_2_, 0.75 mM MgSO_4_, 0.50 mM KH_2_PO_4_, 50 μM KCl, 50 μM H_3_BO_3_, 10 μM MnSO_4_, 2.0 μM ZnSO_4_, 1.5 μM CuSO_4_, 0.075 μM (NH_4_)_6_Mo_7_O_24_, and 72 μM Fe-EDTA. At preparation, the pH and the electrical conductivity (EC) values were 6.0 and 0.6 dS m^–1^, respectively, whereas the iodine concentration in the nutrient solution was below the detection limit of 8 nM, as determined by ICP-MS analysis. The technical peculiarities of each experiment are described in the devoted sections.

### Phenotypical Determinations

Two separate experiments were performed. In both experiments, Arabidopsis plants were initially fed with the base nutrient solution. After 15 days of growth, plants homogeneous in size and leaf number were selected, grouped, and fed with different concentration and/or type of halogen-containing salts added to the nutrient solution. Plants were distributed in nine separate hydroponic trays (three different trays/treatment), and a total number of 90 plants were cultivated for each experimental condition (30 plants/tray).

In the first experiment (exp. 1—phenotype), 0.20 or 10 μM KIO_3_ was added to the nutrient solution. One month later, during the formation/elongation of the main inflorescence, half of the plants (15 plants/tray) was harvested and characterised according to the main morphological traits, such as rosette and inflorescence fresh weight (FW), dry weight (DW), dry matter content, rosette diameter, and inflorescence length. The remaining half was allowed to complete the growing cycle and was characterised in terms of flowering and seed production. Flowering, defined as the presence of the first open flower on the stem, was recorded at intervals of 3 days and expressed on a percentage basis. The percentage of bloomed plants/tray was calculated at each assessment date. Toward the final part of the plants’ life cycle, a periodical harvesting of the produced/matured seeds was carried out until the complete plant desiccation. Seed production was determined in terms of total seed weight/tray (15 plants/tray), number of seeds/silique and number of siliques/plant.

In the second experiment (exp. 2—phenotype), plants were treated by adding either KI, NaI or KBr (0, 10, or 30 μM) to the nutrient solution. Fifteen days after the salt treatment, half of the plants was characterised in terms of plant FW, DW and dry matter content, while the other half was subsequently characterised in terms of flowering (recorded with intervals of 2 days), as described for experiment 1-phenothype.

### Total RNA Extraction and Processing

Gene expression analysis was performed on 3-week-old Arabidopsis plants hydroponically grown on the base nutrient solution (control plants) or in the same medium to which 10 μM of KBr, NaI, or KI was added. Plant material was collected 48 h after the beginning of the treatment. Each sample consisted of a pool of rosettes or roots sampled from three different plants, which were immediately frozen in liquid nitrogen and stored at −80°C until further analysis.

Total RNA from rosettes was extracted as described by Perata et al. (1997), avoiding the use of aurintricarboxylic acid. RNA from roots was extracted using the Spectrum^TM^ Plant Total RNA Kit (Sigma-Aldrich). RNA was subsequently processed for microarray and qPCR analysis. The TURBO DNA-free kit (Thermo Fisher Scientific) was used to remove DNA contaminations and the iScript TM cDNA synthesis kit (Bio-Rad Laboratories) was used for RNA reverse-transcription.

### Microarray Analysis

RNA from rosettes and roots was processed and hybridised to Affymetrix GeneChip Arabidopsis ATH1 Genome Arrays as described by Loreti et al. (2005). Normalisation was performed using Microarray Suite 5.0 (MAS5.0). Differentially expressed genes (DEGs) were selected based on the two following criteria: log_2_FC treated/control ≥ 2 and mas5-Detection *p* ≤ 0.05. In addition, the absolute expression level ≥ 100 mas5-Signal was chosen to select only well-expressed genes. Rosette and root DEGs resulting from KI, NaI, and KBr treatments were processed and visualised in a Venn diagram. Only DEGs commonly regulated by KI- and NaI-treated plants and not responding to KBr treatments were considered specifically linked with the iodine treatment. This group of DEGs was then subjected to gene set enrichment using Gorilla^1^ and analysed with Mapman^2^, whereas the co-expression analysis was performed using Genevestigator^3^.

### Gene Expression Analysis (RT-qPCR)

Quantitative PCR (ABI Prism 7300 Sequence Detection System, Applied Biosystems) was performed using 30 ng cDNA and the iQ SYBR Green Supermix (Bio-Rad Laboratories). *UBIQUITIN10* (*At4g05320*) and *TIP4* (*At2g25810.1*) were used as reference genes. Relative expression levels were calculated using GeNorm^4^. The list of the primers and their sequences are reported in Supplementary Table S1. Four biological replicates were analysed, each consisting of a pool of rosettes or roots sampled from three different plants.

### Feeding With Radioactive Iodine

Two separate experiments were performed by feeding radioactive iodine (^125^I—NaI, PerkinElmer) to hydroponically grown *Arabidopsis thaliana* (exp. 1—radioactive) or tomato, lettuce, wheat and maize (exp. 2—radioactive) plants. Treatments were performed on 1-month-old plants. The solution of ^125^I was prepared by dissolving 60 μl of the commercial radioactive ^125^I product (2.4 mCi/100 μl—9.41 μM) in 250 ml of base nutrient solution. Plants were individually transferred into plastic tubes and treated with the hydroponic solution (with or without Na^125^I). Sampling was performed after 48 h of incubation by collecting leaf and root material, which was immediately frozen in liquid nitrogen, and stored at −80°C until the analysis. Control, non-treated plants (no ^125^I added during their growth) were used in both experiments.

### Protein Extraction, Electrophoresis, and Gel Autoradiography

Leaf and root samples from ^125^I-fed and control plants were ground to fine powder in liquid nitrogen. The protein extraction buffer (50 mM TrisHCl, pH 7.0, 1% w/v SDS, P9599 protease inhibitor cocktail, Sigma-Aldrich) was added to the powder. The resulting solution was vortexed vigorously, and then centrifuged (18,407 *g*, 30 min, 4°C). Radioactive iodine solution (10 μl; prepared as described above) was added to the control samples during the extraction process to check for the occurrence of false positive signals (technical artifacts), possibly due to unspecific binding of iodine with the protein extract.

Protein extracts were dissolved in a 5 × Laemmli buffer, treated at 95°C for 10 min, and a volume of 20 μl (corresponding, approximately, to 65 or 20 μg of proteins, in shoot and root samples, respectively) was loaded to Invitrogen NuPAGE gels (10% Bis-Tris Midi Gels, Thermo Fisher Scientific), together with a protein marker (Precision Plus Protein^TM^ Dual Color Standards, Bio-Rad). After electrophoresis, the gel was rinsed in MilliQ water, and the proteins were fixed (40:7:53 ethanol/glacial acetic acid/H_2_O – 30 min) and then stained (EZ Blue Gel Staining reagent, Sigma-Aldrich – 30 min) on an orbital shaker. After rinsing, gels were exposed to a multipurpose phosphor storage screen (Cyclone Storage Phosphor System, PerkinElmer) in order to obtain a digital image of the radioactivity distribution. Radioactive signals were quantified after 72 h of gel exposure using a Cyclone Phosphor Imaging System (PerkinElmer). In order to prevent the occurrence of any radioactive emissions from the control samples, after each image acquisition, gels were re-exposed for 15 days, and the absence of ^125^I labelled bands was verified in the newly acquired images.

### Database Search for Iodinated Peptides in Protein Datasets From Proteomic Data Repositories

Mass spectrometry data were downloaded from the PRIDE (PRoteomics IDEntification database) archive^5^ (Perez-Riverol et al., 2019). The PRIDE archive was searched to select *A. thaliana* datasets based on the analysis of specific plant organs, such as cauline, rosette and roots, and/or subcellular districts, such as chloroplasts and mitochondria. Datasets were excluded if enrichment/immunopurification strategies were used during protein purification. Finally, 21 experimental sets of nano-LC-ESI-MS/MS raw data included in 14 PRIDE repositories (March 2020) were obtained and re-analyzed by database searching. Only raw files corresponding to the analysis of control/non-treated plants were downloaded and the experimental protocols and the search parameters for each different dataset were annotated. Supplementary Table S2 lists the experimental sets, with details on the MS instrument, plant organ and/or subcellular compartment, sample preparation, and proteomic strategies adopted.

Raw files were searched separately using Proteome Discoverer 2.4 (Thermo Fisher Scientific, United States) with the Mascot v. 2.6 search engine (Matrix Science Ltd., United Kingdom) against the TAIR10 database^6^ (71,567 sequences, accessed May 2017) and a database containing common laboratory contaminants on the MaxQuant website^7^. Workflows were built for each experimental dataset, considering the specific mass tolerance values used for the original search and reported on the PRIDE repository, or in the publication associated with the dataset.

For all the workflows, Cys carbamidomethylation was set as a fixed modification, while iodination at Tyr and His (Δm = +125.8966 Da), oxidation at Met, protein N-terminal acetylation, deamidation at Asp, and pyroglutamate formation at N-terminal Gln were selected as variable modifications. Isotopic labelling was also considered in the modification parameters when performed for protein quantification.

Trypsin was selected as the proteolytic enzyme and peptides were allowed to have a maximum of two missed cleavages. The minimum peptide length was set at six amino acids. The site probability threshold for peptide modification was set at 75. Only high confidence peptide identifications were retained by setting the target false discovery rate (FDR) for PSM at 0.01 and further filtered to keep only peptides (*P* < 0.05) with a Mascot Ion score > 30. In addition, the results of the identification analysis were processed by putting together the output of iodinated peptides from all the datasets and further applying a filter to keep only those identified with a Mascot Ion score > 50, in at least one dataset, to limit the identification to peptides with the best scoring matches and corresponding to high certainty. The presence of the MS/MS spectrum of the unmodified counterpart was verified for each iodinated peptide to further validate the identification.

### Protein Bioinformatics

Proteins containing iodinated peptides were functionally annotated according to MapMan categories by using the Mercator pipeline^8^. Final outputs were integrated with data from the available literature. Protein interaction networks were obtained with STRING v. 11^9^, which was also used to provide information on known gene ontology categories. Venn diagrams were depicted using a web tool at http://bioinformatics.psb.ugent.be/webtools/Venn. The Protein Abundance Database (PAXdb) was also queried to evaluate quantitative levels of modified *A. thaliana* proteins at https://pax-db.org.

### Statistical Analysis

Data concerning phenotypical determinations and qPCR-based gene expression analysis were analysed by one-way ANOVA coupled with the LSD *post hoc* test, when they followed a normal distribution and there was homogeneity of variances. When one of these two prerequisites was violated, a Kruskal-Wallis test for non-parametric statistic was performed and the significance letters were graphically assigned using a box-and-whisker plot with a median notch. Significant differences between the means (*P* < 0.05) are indicated by different letters in the figures and tables.

## Results

### Effects of Iodine on the Plant Phenotype

**FIGURE 1:**
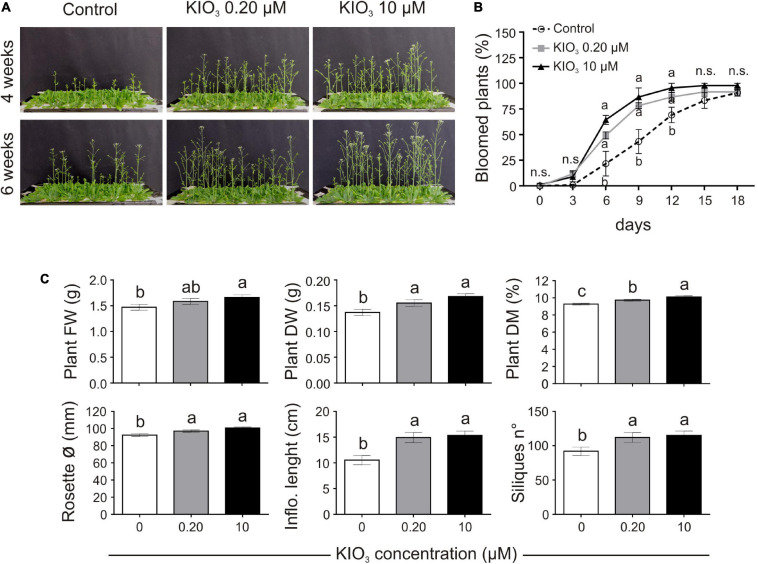
Impact of iodine on plant growth and development (exp. 1-phenotype). **(A)** Lateral view of plants after 4 or 6 weeks from the onset of KIO_3_ treatment. **(B)** Flowering time curve; the percentage of bloomed plants/tray was calculated every 3 days after the opening of the first flower (day 0). **(C)** Morphological data on plant FW, DW, dry matter content, rosette diameter, inflorescence length and number of produced siliques/plant, determined 1 month after the onset of KIO_3_ treatments. Values indicated by different letters significantly differ from each other (according with one-way ANOVA, LSD *post hoc* test, *P* ≤ 0.05). In particular, the statistical analysis of flowering **(B)** was performed by comparing the percentage of bloomed plants of each tray (considered as biological replicates) within each sampling point. When data followed a Normal distribution and there was homogeneity of variances, they were subjected to one-way ANOVA and values indicated by different letters significantly differ from each other (LSD *post hoc* test, *P* ≤ 0.05). When one of this two prerequisite was violated, a Kruskal-Wallis test was performed. Error bars (±SE) are shown in graphs.

The effects of low amounts of KIO_3_ (0.20 or 10 μM) on hydroponically grown Arabidopsis plants, compared to plants cultivated on a control nutrient solution, were evaluated in terms of plant morphology, biomass, and seed production (exp. 1-phenotype). No phytotoxicity symptoms were observed on plants and the most significant phenotypical effect was a delay of flowering in the control plants, compared to KIO_3_ (either 0.20 or 10 μM) (Figures 1A,B). Twelve days after the opening of the first flower, plants treated with 0.20 or 10 μM KIO_3_ were close to complete flowering, as 87 and 96% of the plants had bloomed, respectively, vs. 69% of the control plants (Figure 1B). Control plants took about 18 days to complete blooming.

Plant biomass, evaluated 1 month after the addition of KIO_3_ to the nutrient solution, was significantly lower in control plants, both in FW and DW (Figure 1C). When compared to the control, the plant FW increased by approximately 7.7 and 13% with addition of 0.20 and 10 μM KIO_3_ in the nutrient solution, respectively, whereas the DW increased by 13 and 22%, respectively. The effect on plant FW was mostly ascribable to the inflorescence, as no significant differences were evident in terms of the rosette FW values (Supplementary Table S3). The concentration of iodine in the nutrient solution had a marked effect on the inflorescence length, which was approximately 41 and 45% longer compared to the control in 0.20 and 10 μM KIO_3_ treated plants, respectively (Figure 1C), and a comparable effect was seen on the inflorescence FW and DW (Supplementary Table S3). Additionally, the rosette diameter in the control was smaller, and the application of 0.20 or 10 μM KIO_3_ increased it by approximately 5 and 9%, respectively (Figure 1C). The plant dry matter content positively correlated with the increased iodate concentrations (Figure 1C).

Seed production was determined in terms of total seed weight, seeds/silique and number of siliques/plant. The number of seeds contained in each silique was not affected by iodate treatments (Supplementary Table S3), whereas the number of siliques produced by each plant was lower in the control, compared to the addition of both 0.20 and 10 μM KIO_3_ (Figure 1C). This influenced the total seed production, which, 1 month after the addition of KIO_3_ to the nutrient solution, was much higher in plants treated with iodate (more than 50 and 35%, respectively, in 0.20 and 10 μM KIO_3_ treated plants in comparison with the control) (Supplementary Table S3).

**FIGURE 2:**
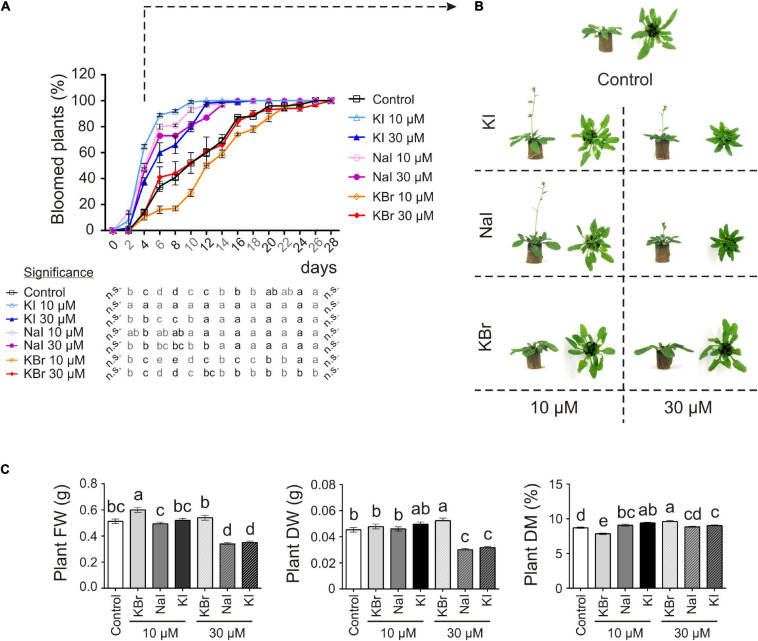
Impact of iodine on plant growth and development (exp. 2-phenotype). **(A)** Flowering curve; the percentage of bloomed plants/tray was calculated every 2 days after the opening of the first flower (day 0). **(B)** Representative control, and KI-, NaI- or KBr-treated plants (10 and 30 μM) after 15 days from the onset of the treatments. Pictures were taken after 4 days from the opening of the first flower on the main stem. **(C)** Morphological data on plant FW, DW, dry matter content determined 15 days after the onset of the treatments. Values indicated by different letters significantly differ from each other (according with one-way ANOVA, LSD *post hoc* test, *P* ≤ 0.05). In particular, the statistical analysis of flowering **(A)** was performed by comparing the percentage of bloomed plants of each tray (considered as biological replicates) within each sampling point. When data followed a Normal distribution and there was homogeneity of variances, they were subjected to one-way ANOVA and values indicated by different letters significantly differ from each other (LSD *post hoc* test, *P* ≤ 0.05). When one of this two prerequisite was violated, a Kruskal-Wallis test was performed. Error bars (±SE) are shown in graphs.

Adding exogenous iodine in the form of KIO_3_ countered the delay in flowering of control plants (Figures 1A,B). This was confirmed in experiment 2 (exp. 2-phenotype), when iodine was added in the form of KI or NaI (Figures 2A,B). The possible effects of potassium or bromide, as an alternative halogen, were evaluated and then ruled out, as a similar behaviour was observed in plants treated with KI or NaI, but not with KBr (Figures 2A,B).

The application of 10 μM KI and NaI promoted flowering, without negatively impacting the plant biomass production (Figure 2C), whereas 30 μM KI or NaI reduced plant growth (Figure 2C), although the promoting effect of iodine on flowering was still present (Figures 2A,B). Four days after the opening of the first flower (day 0), more than 50% of KI- and NaI-treated plants had bloomed vs. 14% of the control plants and 10% and 14% of the 10 and 30 μM KBr-treated plants, respectively. Moreover, the floral transition was almost complete in 10 μM KI- and NaI-fed plants in the subsequent 6 days (10 days after day 0). Two and four more days were required for 30 μM KI- and NaI-fed plants, respectively (12 and 14 days after day 0), whereas the control and KBr-treated plants completed blooming in the subsequent 18 days (22 days after day 0) (Figure 2A).

### Effects of Iodine on Gene Expression

The response of plants to iodine was analyzed at the transcriptomic level. To identify genes whose expression was specifically altered by iodine, Arabidopsis plants were treated by adding 10 μM of NaI, KI, or KBr to the nutrient solution, compared to the untreated control plants. The resulting RNAs were analyzed by hybridisation on ATH1 microarrays. To rule out the possible generic effects of halogens, we searched the microarray dataset for genes that responded to KI and NaI, but not to KBr. In addition, a comparison between KBr- and KI-treated plants enabled us to rule out the possible transcriptional regulation of genes exerted by potassium, as K^+^ ion was common to both salts.

**FIGURE 3:**
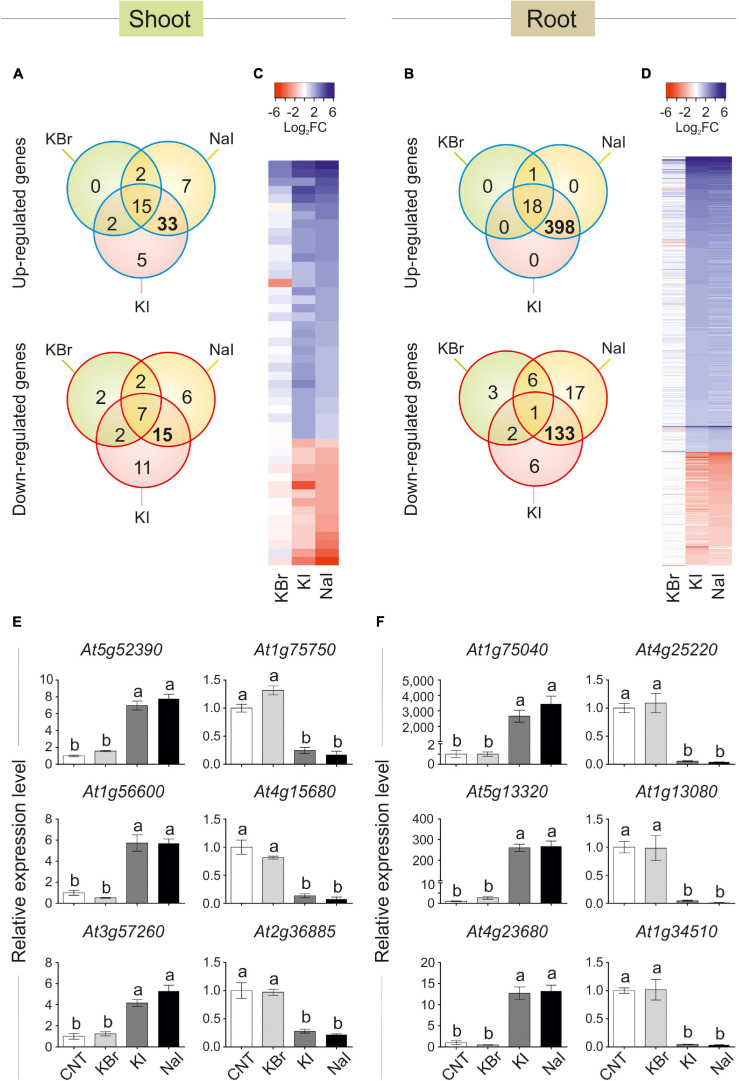
Transcriptional regulation of gene expression induced by iodine. Venn diagram showing the number of genes differentially regulated in shoot **(A)** or root **(B)** tissues of KBr-, NaI-, and KI-treated plants (10 μM—48 h), when compared with the control. Heatmap showing the pattern of expression of the genes analysed in the shoot **(C)** or root **(D)** tissues in response to NaI, KI or KBr treatments, when compared with the control. qPCR validation of selected genes up- or down-regulated by iodine treatments (commonly regulated by NaI and KI, but not KBr) in shoot **(E)** or root **(F)** tissues. qPCR data are mean ± SE of four biological replicates, each composed of a pool of three different rosettes. Values indicated by different letters significantly differ from each other (according with one-way ANOVA, LSD *post hoc* test, *P* ≤ 0.05).

Data visualisation with a Venn diagram showed that several genes were specifically regulated by iodine, as up- or down-regulated genes in both NaI- and KI- but not in KBr-treated plants were 33 (51% of DEGs) and 15 (33% of DEGs) in the shoot (Figure 3A), and 398 (95% of DEGs) and 133 (79% of DEGs) in the root (Figure 3B), respectively. The similarity and specificity in the expression pattern of KI- and NaI-treated plants were confirmed by the heatmaps generated from the analysis of the shoot (Figure 3C) and root (Figure 3D) expression data.

To validate the microarray analysis, a subset of three I^–^-induced and three I^–^-repressed genes were analysed by qPCR, corroborating the specific regulation of iodine on their expression in both shoot (Figure 3E) and root (Figure 3F) samples.

The complete list of the KI and NaI commonly and not responding to KBr up- and down-regulated genes is reported in Supplementary Tables S4, S5 (shoot tissue), and Supplementary Tables S6, S7 (root tissue), respectively.

The polypeptides codified by the iodine-regulated genes did not show a preferential site of action in the cell, as their predicted localisations include cytoplasm, chloroplast, cell wall, nucleus, mitochondrion, vacuole and apoplast (Supplementary Tables S4–S7).

**FIGURE 4:**
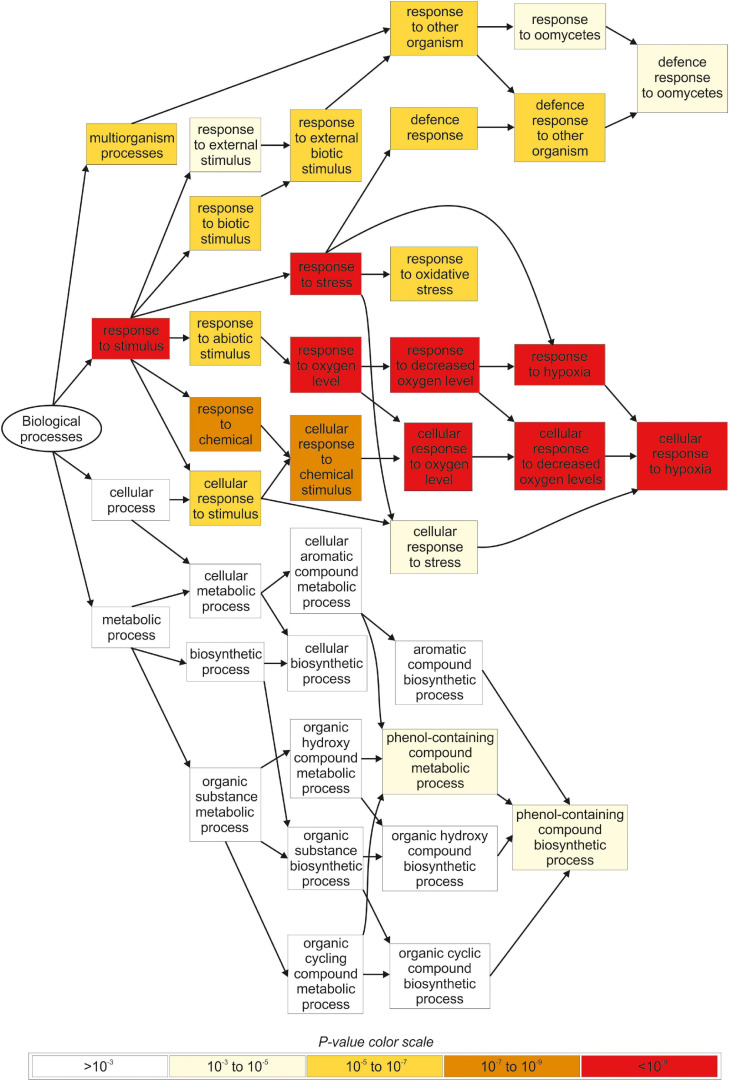
Overview of the main biological processes affected by iodine based on the GO terms enrichment analysis carried out in root tissues. Only genes regulated in NaI- and KI-treated plants, and not in KBr-treated plants, when compared with the control, were analysed. The figure was extracted from GOrilla (http://cbl-gorilla.cs.technion.ac.il) and reproduced. In this analysis, DEGs with log_2_FC ≥ 2.5 or log_2_FC ≤ –2.5 were used.

The gene ontology (GO) analysis identified several functional categories regulated by iodine in the roots (Figure 4, Supplementary Figure S2 and Supplementary Tables S8, S9), whereas no statistically significant GO terms were identified by analysing the DEG data on the shoots.

The most representative biological processes affected by iodine in the roots were related to the *response to stimulus* (GO:0050896), and the downstream categories associated with *response to abiotic* (GO:0009628) and *biotic stimulus* (GO:0009607) (Figure 4 and Supplementary Table S8). The main molecular functions regulated by iodine in the roots were related to *antioxidant* (GO:0016209) and *oxidoreductase activity* (GO:0016491) and related child terms, in particular *peroxidase activity* (GO:0004601) and *oxidoreductase activity*, *acting on peroxide as acceptor* (GO:0016684) (Supplementary Figure S2 and Supplementary Table S9).

DEGs analysis performed with MapMan highlighted an over-representation of several genes in root samples that were related to calcium regulation and protein modification/degradation (Supplementary Figure S3A), together with genes encoding for the large enzyme families including peroxidases, oxidases, glutathione S-transferases, and cytochrome P450 (Supplementary Figure S3B).

The relatively low number of genes regulated by iodine in the shoots prevented a gene ontology analysis from being performed. However, in terms of the most well characterised genes specifically regulated by iodine treatments in the shoot, the main pathways affected were directly or indirectly involved in biotic (approximately 48 or 40% of up- or down-regulates genes, respectively) or abiotic (approximately 45 or 33% of up- or down-regulates genes, respectively) stress response pathways (Supplementary Tables S4, S5). Several genes playing a role in the transition to flowering (*At4g19191* and *At1g75750*) and embryo and pollen development (i.e., *At1g21310*, *At3g54150*) are also worth mentioning.

The involvement of iodine in the defence response, highlighted by the previous analyses performed on root samples, was also suggested by querying all publicly available microarray datasets (see footnote) using the list of iodine-responsive genes of both shoot (Supplementary Figure S4) and root (Supplementary Figure S5) tissues. The majority of the up- or down-regulated genes were commonly modulated by the presence of fungal infection, salicylic acid (SA) or synthetic analogues of SA, such as benzothiadiazole (Kouzai et al., 2018).

### Protein Iodination in Plants

Iodine can be found in plant tissues not only in a mineral form but also in organic compounds (Wang et al., 2014; Smoleń et al., 2020). To verify the possible *in vivo* incorporation of iodine into proteins, we carried out two different experiments by feeding hydroponically grown plants with ^125^I and carrying out the autoradiography of the SDS-PAGE of the relative protein extracts to detect possible radio-labelled proteins. The experiments were performed first with Arabidopsis plants, and then with other species, namely maize, tomato, wheat and lettuce.

**FIGURE 5:**
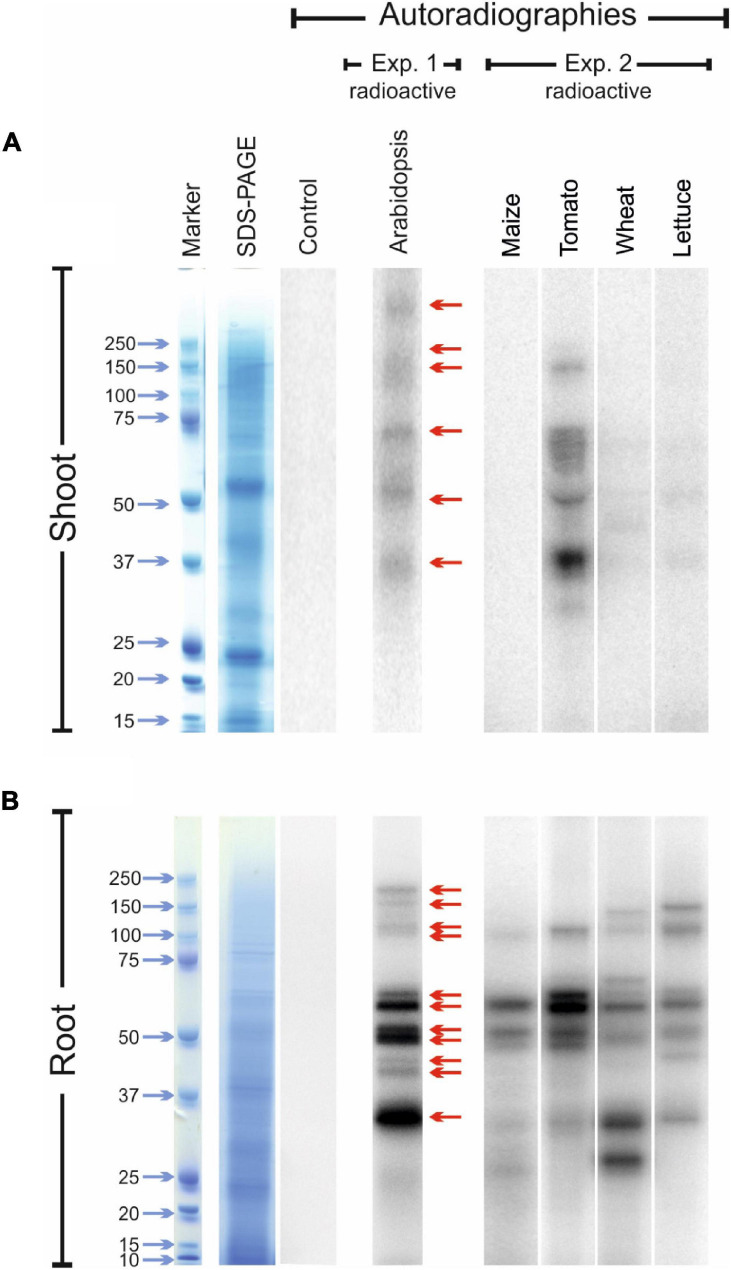
Autoradiographies of the SDS-PAGE gels. Comparison between the position and relative intensities of ^125^I radiolabelled bands of representative shoot **(A)** and root **(B)** protein extracts from ^125^I treated Arabidopsis (exp. 1-radioactive), and maize, tomato, wheat and lettuce plants (exp. 2-radioactive). Sampling was performed after 48 h of ^125^I incubation. In both the experiments, autoradiographies were acquired after 72 h of gel exposition to the multipurpose phosphor storage screen. Representative pictures of total stained protein extracts (SDS-PAGE) and of the autoradiographies of control samples after 15 days of exposition are also shown. Controls consisted in protein extracts obtained from plants untreated with ^125^I during their growth, to which the radioactive solution containing ^125^I was added during the extraction process.

The experiment carried out with Arabidopsis plants revealed the presence of at least six radio-labelled bands at different molecular mass values in the protein extracts from shoot tissues (Figure 5A; exp. 1-radioactive) and eleven radio-labelled bands from root tissues (Figure 5B; exp. 1-radioactive), indicating the presence of proteins likely containing iodo-amino acids. Iodinated proteins were preferentially present in root tissues, as the abundance and intensity of ^125^I-labelled bands were higher in the root than in the shoot extracts. No radioactive signals were observed in the shoot and root control samples (samples added with ^125^I solution during protein extraction).

Several ^125^I -labelled bands were also observed in the leaf extracts of tomato, wheat and lettuce samples, whereas no ^125^I-containing bands were visible in the leaf protein extracts of maize (Figure 5A; exp. 2-radioactive). A clear radioactive signal was detected in several root proteins extracted from all the species analysed, including maize (Figure 5B; exp. 2-radioactive). Also in this case, the intensity of the radiolabelled bands was higher in root than in shoot extracts. A good degree of conservation of the molecular mass values of the putatively iodinated proteins was observed among the five plant species analysed (Figure 5).

### Identification of Iodinated Proteins in *Arabidopsis thaliana*

The identification of the radiolabelled proteins described above was hampered by the presence of a radioactive isotope, which meant that our samples did not meet the safety rules for proteomic facilities. To maximise the probability of success in identifying targets of protein iodination, we then focused on the nano-LC-ESI-MS/MS raw data already acquired within the framework of experimental studies on different organs/subcellular districts of *Arabidopsis thaliana*, and released in the public repository PRoteomics IDEntification Database (PRIDE) Archive (Perez-Riverol et al., 2019).

The datasets considered for our analysis refer to many different experimental conditions in terms of plant growth, treatment, and cultivation regimen, as well as sample processing and fractionation performed before proteomic analysis. No experiments were explicitly related to iodination studies; the presence of iodine occurred accidentally, as a consequence of its natural presence in the cultivation environment (i.e., soil, air, irrigation water), or because it was conventionally present in the MS growing medium (Murashige and Skoog, 1962), which is widely used in studies based on *in vitro* plant tissue culture.

In proteins, iodination affects various amino acids, depending on the reaction conditions (Ramachandran, 1956), but generally following the reactivity order Tyr >> His ≥ Trp > Cys.

Mono-iodination at Tyr and His residues were thus considered in the searching parameters as variable modifications. The output of the database search, in terms of proteins iodinated at Tyr or His residues has been reported in Supplementary Table S10.

The iodinated peptides were identified in 16 out of the 21 datasets analysed. A total of 106 iodinated peptides, corresponding, respectively, to 42 and 40 protein accessions in the TAIR10 database of *A. thaliana* leaves (chloroplast, cauline, and rosette) (Table 1) and roots (Table 2), were identified. Iodinated sequences differently modified for deamidation, and/or protein N-terminal acetylation, and/or Met oxidation are reported as a unique iodinated peptide inventory.

**TABLE 1:** Iodinated peptides identified in *A. thaliana* leaves (chloroplast, cauline, and rosette) by database searching of mass spectrometric data retrieved from PRIDE repository.

Protein accession	Description	Iodinated sequence	Iodinated site	DATASET
ATCG00120.1	ATP synthase subunit alpha	EAYPGDVFYLHSR	[Y3]	ChlorBN
		SVYEPLQTGLIAIDSMIPIGR	[Y3]	ChlorBN
ATCG00480.1	ATP synthase subunit beta	GIYPAVDPLDSTSTMLQPR	[Y3]	ChlorBN
		GSITSIQAVYVPADDLTDPAPATTFAHLDATTVLSR	[Y10]	ChlorBN
		IVGEEHYETAQQVK	[Y7]	ChlorBN, Chlo3516
		VALVYGQMNEPPGAR	[Y5]	ChlorBN
		VGLTALTMAEYFR	[Y11]	ChlorBN
AT4G04640.1	ATPase, F1 complex, gamma subunit protein	GLGLEYTVISVGK	[Y6]	ChlorBN
AT1G29910.1	Chlorophyll A/B binding protein 3	YLGPFSGESPSYLTGEFPGDYGWDTAGLSADPETFAR	[Y1]*	ChlorBN
AT1G20340.1	Cupredoxin superfamily protein	NNAGYPHNVVFDEDEIPSGVDVAK	[H7]*	CAU, Ros
AT5G66190.1	Ferredoxin-NADP(+)-oxidoreductase 1	LVYTNDGGEIVK	[Y3]	ChlorBN
AT4G38970.1	Fructose-bisphosphate aldolase 2	ATPEQVAAYTLK	[Y9]	ChlorBN
AT5G42270.1	FtsH extracellular protease family	DYSMATADVVDAEVR	[Y2]	ChlorBN
AT3G09260.1	Glycosyl hydrolase superfamily protein	CSSYVNAK	[Y4]	ChlorBN
		GPALWDIYCR	[Y8]	ChlorBN
		FGLYYVDFK	[Y4]*	ChlorBN
AT4G10340.1	Light harvesting complex of photosystem II 5	SEIPEYLNGEVAGDYGYDPFGLGK	[Y15]*	ChlorBN
		TGALLLDGNTLNYFGK	[Y13]	ChlorBN
AT5G54270.1	Light-harvesting chlorophyll B-binding protein 3	YLGPFSVQTPSYLTGEFPGDYGWDTAGLSADPEAFAK	[Y21]*	ChlorBN
AT2G34430.1	Light-harvesting chlorophyll-protein complex II subunit B1	YLGPFSGEPPSYLTGEFPGDYGWDTAGLSADPETFAR	[Y12]*	ChlorBN, PXD010730, LFD
AT2G24940.1	Membrane-associated progesterone binding protein 2	SFYGSGGDYSMFAGK	[Y3]	Ros
AT4G22890.5	PGR5-LIKE A	FLEASMAYVSGNPILNDEEYDKLK	[Y20]	ChlorBN
ATCG00540.1	Photosynthetic electron transfer A	GGYEITIVDASNGR	[Y3]	ChlorBN
AT4G03280.1	Photosynthetic electron transfer C	GDPTYLVVENDK	[Y5]	ChlorBN
AT4G28750.1	Photosystem I reaction centre subunit IV/PsaE protein	VNYANISTNNYALDEVEEVAA	[Y3]	ChlorBN
AT2G20260.1	Photosystem I subunit E-2	VNYANISTNNYALDEVEEVK	[Y3]	ChlorBN
AT1G31330.1	Photosystem I subunit F	LYAPESAPALALNAQIEK	[Y2]	ChlorBN, Ros
AT1G52230.1	Photosystem I subunit H2	SVYFDLEDLGNTTGQWDVYGSDAPSPYNPLQSK	[Y19]	ChlorBN
ATCG00340.1	Photosystem I, PsaA/PsaB protein	TSYGFDVLLSSTSGPAFNAGR	[Y3]	ChlorBN
AT1G03600.1	Photosystem II family protein	DIYSALNAVSGHYVSFGPTAPIPAK	[Y3]	ChlorBN
AT1G06680.1	Photosystem II subunit P-1	SITDYGSPEEFLSQVNYLLGK	[Y5]*	ChlorBN, PXD010730
AT1G79040.1	Photosystem II subunit R	YGANVDGYSPIYNENEWSASGDVYK	[Y12]*	ChlorBN
AT2G05100.1	Photosystem II light harvesting complex gene 2.1	STPQSIWYGPDRPK	[Y8]	ChlorBN
		YLGPFSENTPSYLTGEYPGDYGWDTAGLSADPETFAK	[Y12]*	ChlorBN
AT2G20890.1	Photosystem II reaction centre PSB29 protein	AYIEALNEDPK	[Y2]	ChlorBN
AT2G30790.1	Photosystem II subunit P-2	SITDYGSPEQFLSQVNYLLGK	[Y5]*	ChlorBN
AT3G50820.1	Photosystem II subunit O-2	GGSTGYDNAVALPAGGR	[Y6]	ChlorBN, Chlo3516
AT4G05180.1	Photosystem II subunit Q-2	YYSETVSSLNNVLAK	[Y2]	ChlorBN
AT4G21280.1	Photosystem II subunit QA	LFDTIDNLDYAAK	[Y10]	ChlorBN
		YYAETVSALNEVLAK	[Y2]*	ChlorBN
ATCG00020.1	Photosystem II reaction centre protein A	ETTENESANEGYR	[Y12]	ChlorBN, TMAR
		FGQEEETYNIVAAHGYFGR	[Y8]	ChlorBN
ATCG00270.1	Photosystem II reaction centre protein D	AAEDPEFETFYTK	[Y11]	ChlorBN
ATCG00280.1	Photosystem II reaction centre protein C	DIQPWQERRSAEYMTHAPLGSLNSVGGVATEINAVNYVSPR	[H16]	LFD
		RSAEYMTHAPLGSLNSVGGVATEINAVNYVSPR	[H8]	CAU, Ros
		SAEYMTHAPLGSLNSVGGVATEINAVNYVSPR	[H7]*	ChlorBN, LFP
ATCG00680.1	Photosystem II reaction centre protein B	LAFYDYIGNNPAK	[Y4]*	ChlorBN, TMAR, CAU, Ros
		YQWDQGYFQQEIYR	[Y7]*	ChlorBN
AT5G07020.1	Proline-rich family protein	AVDYSGPSLSYYINK	[Y12]	ChlorBN
AT1G74470.1	Pyridine nucleotide-disulphide oxidoreductase	SIDAGDYDYAIAFQER	[Y7]	ChlorBN
ATMG00280.1	Ribulose bisphosphate carboxylase large chain, catalytic domain	GGLYFTKDDENVNSQPFMR	[Y4]	CLLF, LFD, LFP, LFPT
AT1G67090.1	Ribulose bisphosphate carboxylase small chain 1A	EYPNAFIR	[Y2]	ChlorBN, CAU, Ros
ATCG00490.1	Ribulose-bisphosphate carboxylases	GHYLNATAGTCEEMIKR	[Y3]*	ChlorBN, Chlo10545,CAU
		LTYYTPEYETKDTDILAAFR	[Y8]*	CAU, Ros
AT1G71500.1	Rieske (2Fe-2S) domain-containing protein	SPAEGAYSEGLLNAR	[Y7]	ChlorBN
AT2G39730.1	Rubisco activase	GLAYDTSDDQQDITR	[Y4]	ChlorBN
AT1G54780.1	Thylakoid lumen 18.3 kDa protein	ADAFEYADQVLEK	[Y6]	ChlorBN
		ETYVVDDAGVLSR	[Y3]	ChlorBN

*Protein Accession and Description, sequence of iodinated peptide and iodination site have been reported. In the case of peptide sequences in which more than one iodinated residues were identified, the asterisk indicates the iodination site identified with the highest score. The datasets in which the iodinated peptides were identified have been indicated.*

**TABLE 2:** Iodinated peptides identified in *A. thaliana* roots by database searching of mass spectrometric data retrieved from PRIDE repository.

Protein accession	Description	Iodinated sequence	Iodinated site	DATASET
AT5G09810.1	Actin 7	NYELPDGQVITIGAER	[Y2]	Root
AT1G28290.1	Arabinogalactan protein 31	NGYFLLLAPK	[Y3]	RT
AT5G08680.1	ATP synthase alpha/beta family protein	VGLTGLTVAEYFR	[Y11]	Root
AT1G45130.1	Beta-galactosidase 5	YDEDIATYGNR	[Y1]	RT, RTTP, RTUZ
AT2G43610.1	Chitinase family protein	YCSPSTTYPCQPGK	[Y1]	Root
AT3G43670.1	Copper amine oxidase family protein	GTAYENVEDLGEK	[Y4]	RT, RTUZ
AT1G78850.1	D-mannose binding lectin protein	TGDSSLVAYVK	[Y9]	Root, RT, RTTP
AT5G20080.1	FAD/NAD(P)-binding oxidoreductase	IFYTVDNPTK	[Y3]	Root
AT4G20830.1	FAD-binding Berberine family protein	DVDIGVNDHGANSYK	[Y14]	RTTP
AT5G44380.1	FAD-binding Berberine family protein	YGLAGDNVLDVK	[Y1]	RTUZ
AT5G50950.1	FUMARASE 2	IGYDNAAAVAK	[Y3]	Root
AT3G04120.1	Glyceraldehyde-3-phosphate dehydrogenase C sub1	LKGILGYTEDDVVSTDFVGDNR	[Y7]	RT, RTTP, RTUZ
AT4G16260.1	Glycosyl hydrolase superfamily protein	AFYTNLASR	[Y3]	Root
		LYDPNQAALNALR	[Y2]	Root
AT3G13790.1	Glycosyl hydrolases family 32 protein	HDYYTIGTYDR	[Y4]*	RT, RTUZ
AT3G19390.1	Granulin repeat cysteine protease family protein	VVTIDGYEDVPQNDEK	[Y7]	Root
AT3G12580.1	Heat shock protein 70	NALENYAYNMR	[Y6]	Root
		TTPSYVAFTDSER	[Y5]	Root
AT5G42020.1	Heat shock protein 70 (Hsp 70) family protein	NALETYVYNMK	[Y8]*	Root
AT3G16430.1	Jacalin-related lectin 31	VYVGQAQDGISAVK	[Y2]	Root
AT2G22170.1	Lipase/lipooxygenase, PLAT/LH2 family protein	VYDKYGDYIGIR	[Y8]*	Root
AT3G16460.1	Mannose-binding lectin superfamily protein	IYASYGGEGIQYVK	[Y5]*	Root
AT3G48890.1	Membrane-associated progesterone binding protein 3	MFYGPGGPYALFAGK	[Y3]	Root
AT4G19410.1	Pectinacetylesterase family protein	DITGGSYIQSYYSK	[Y7]	RT
AT4G30170.1	Peroxidase family protein	EVVVLTGGPSYPVELGR	[Y11]	Root, RT, RTUZ
		IYNFSPTTR	[Y2]	Root
		TGFYQNSCPNVETIVR	[Y4]	Root, RT
AT1G05240.1	Peroxidase superfamily protein	GDSDPSMNPSYVR	[Y11]	RT, RTTP, RTUZ
AT2G37130.1	Peroxidase superfamily protein	PTPDPNAVLYSR	[Y10]	RT, RTUZ
		QQVETLYYK	[Y8]	RT, RTTP, RTUZ
AT3G01190.1	Peroxidase superfamily protein	TFDLSYFTLVAK	[Y6]	RT, RTUZ
AT5G17820.1	Peroxidase superfamily protein	DSVALAGGPSYSIPTGR	[Y11]	Root, RT
		VGFYSQSCPQAETIVR	[Y4]	RT
AT1G79550.1	Phosphoglycerate kinase	YSLKPLVPRLSELLGVEVVMANDSIGEEVQK	[Y1]	RT
AT4G20260.4	Plasma-membrane associated cation-binding protein 1	VVETYEATSAEVK	[Y5]	Root
AT2G47540.1	Pollen Ole e 1 allergen and extensin family protein	GISGAILQNYR	[Y10]	Root
AT5G05500.1	Pollen Ole e 1 allergen and extensin family protein	TDSYGHFYGELK	[Y4]	RT, RTTP, RTUZ
AT4G14060.1	Polyketide cyclase/dehydrase and lipid transport superfamily protein	VYDTILQFIQK	[Y2]	Root
		ATSGTYVTEVPLKGSAEK	[Y6]	Root
AT4G23680.1	Polyketide cyclase/dehydrase and lipid transport superfamily protein	VYDVVYQFIPK	[Y2]	Root
AT2G19760.1	Profilin 1	TNQALVFGFYDEPMTGGQCNLVVER	[Y10]	RTTOF
AT4G02270.1	Root hair specific 13	VDAYGNELVPISILSSK	[Y4]	Root, RTTP, RTUZ
AT4G26220.1	S-adenosyl-L-methionine-dependent methyltransferases	GLLKSEELYKYILETSVYPR	[Y9]	RTTP
AT1G58270.1	TRAF-like family protein	FLDSYTSDSFSSGGR	[Y5]	Root
AT1G45201.1	Triacylglycerol lipase-like 1	FVYNNDVVPR	[Y3]	Root
AT3G55440.1	Triosephosphate isomerase	IIYGGSVNGGNCK	[Y3]	Root
AT4G30270.1	Xyloglucan endotransglucosylase/hydrolase 24	NYESLGVLFPK	[Y2]	RT
		LVPGNSAGTVTTFYLK	[Y14]	RTTP

*Protein Accession and Description, sequence of iodinated peptide and iodination site have been reported. In the case of peptide sequences in which more than one iodinated residues were identified, the asterisk indicates the iodination site identified with the highest score. The datasets in which the iodinated peptides were identified have been indicated.*

**FIGURE 6:**
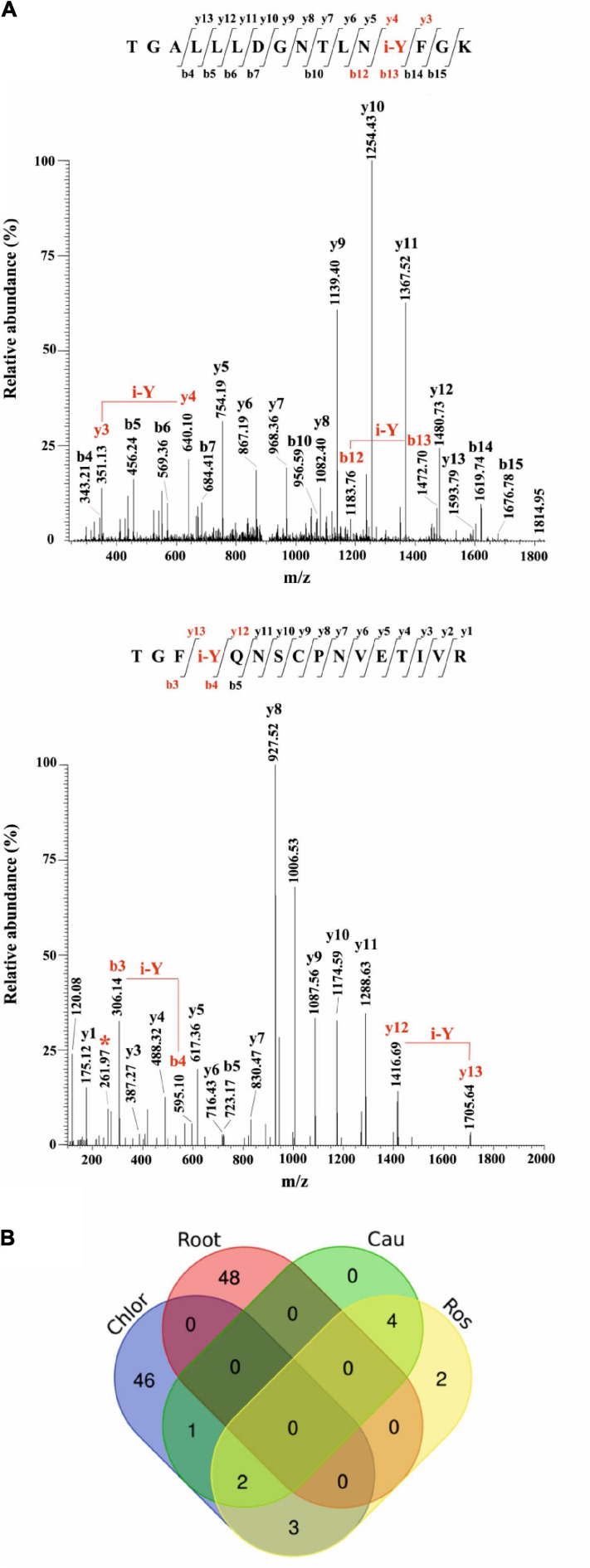
Iodination in *A. thaliana* proteins identified by database searching of nano-LC-ESI-MS/MS raw data from a public repository (PRIDE). **(A)** Unambiguous assignment of iodination sites by MS/MS analysis in two peptides from chloroplast (light harvesting complex of photosystem II 5, AT4G10340.1), upper panel, and roots (peroxidase superfamily protein, AT4G30170.1), lower panel. The peptides are identified by both y, and b ions. Red labels in the spectra evidence the mass shift corresponding to the iodinated tyrosine (i-Y). **(B)** Venn Diagram showing the iodinated peptide sequences identified in the datasets of chloroplasts (Chlor), cauline (Cau), rosette (Ros), and roots (Root).

Most of the modified peptides were found to be iodinated at Tyr residues, while His iodination was identified in only five peptides. Representative MS/MS spectra of Tyr-iodinated peptides are reported in Figure 6A.

To evaluate the entire output of iodinated peptides identified, iodinated sequences for chloroplasts, caulines, rosettes, and roots were processed and visualised in a Venn diagram (Figure 6B). This showed the presence of the common iodinated peptides for the chloroplast cauline and rosette subsets. The root subset was clearly distinct from the other three subsets that were all from the green parts of the plant.

### Iodinated Proteins in *A. thaliana* Leaves

Iodinated peptides identified in 11 datasets of cauline, rosette, and leaf-isolated chloroplasts were assigned to 42 proteins (Supplementary Table S10). Most of the modified species were in the dataset of chloroplastic proteins (Figure 6B). STRING interaction analysis of the modified proteins revealed a single network of 40 iodinated proteins (PPI enrichment *p*< 1.0e^–16^) (Supplementary Figure S6A and Supplementary Table S11). A total of 31 of the 40 proteins in this network were involved in *photosynthesis* (GO:0015979) (Supplementary Table S12), as also attested by their functional analysis, according to MapMan categories (Supplementary Figure S6B and Supplementary Table S13). Moreover, the main molecular functions in which iodinated proteins are involved were related to *chlorophyll binding* (GO:0016168), *protein domain specific binding* (GO:0019904), *tetrapyrrole binding* (GO:0046906), and *electron transfer activity* (GO:0009055) (Supplementary Table S12). In particular, ten proteins were identified in two or more datasets, thus resulting in the most representative targets of the iodination reaction. Some of these proteins were constituents of the photosystem II (PSII), i.e., proteins of the reaction centre (PsbA, PsbB), oxygen evolving centre (PsbO, PsbP) and light harvesting complex II (LHCB1B1), or part of the photosystem I (PSI) (PsaF, PETE2). Three proteins showed ribulose-1,5-bisphosphate carboxylase/oxygenase (RuBisCo) activity and were involved in the Calvin Cycle (RBCL, ORF110A, RBCS1A). These proteins were largely abundant in leaf tissues, especially the PSII component PsbB and RuBisCo large and small chains (RBCL, RBCS1A), according to the data reported in PAXdb.

### Iodinated Proteins in *A. thaliana* Roots

Iodinated peptides identified in 5 datasets of roots were assigned to 40 proteins (Supplementary Table S10). The STRING interaction analysis recognised three networks containing 24 of the 40 iodinated proteins (Supplementary Figure S7A and Supplementary Table S11). The GO analysis for these proteins showed a significant over-representation of biological processes related to the *response to stress* (GO:0006950), *response to oxidative stress* (GO:0006979), *response to toxic substances* (GO:0009636), and *response to stimulus* (GO:0050896). For the molecular functions, the most enriched categories were *copper ion binding* (GO:0005507) and *peroxidase activity* (GO:0004601) (Supplementary Table S14).

The functional analysis of the iodinated proteins in roots, according to MapMan categories, highlighted a broad range of biological roles (Supplementary Figure S7B and Supplementary Table S13). In particular, among the iodinated proteins identified, 12 were found in two or more datasets. Five proteins belonged to the classical plant (class III) peroxidase subfamily (At4g30170, At1g05240, At2g37130, At3g01190, At5g17820). The alignment of the protein sequences of the peroxidases mentioned above showed that iodinated residues in all peptides preferentially corresponded to conserved Tyr residues, while only two iodinated tyrosines were unrelated (Supplementary Figure S8).

The other iodinated proteins included: (i) copper amine oxidase (CUAOy2), a cell-wall oxidase showing primary amine oxidase activity; (ii) beta-galactosidase 5 (BGAL5), a glycoside hydrolase involved in the modification of cell wall polysaccharides; (iii) glycosyl hydrolases family 32 protein (ATBFRUCT1), acting as a cell wall invertase; (iv) Pole Ole1 allergen/extension domain (IPR006041)-containing proline-rich protein-like 1 (PRPL1-MOP10) and root hair specific 13 protein (RHS13), which are cell-wall components; (v) D-mannose binding lectin protein (MBL1); and (vi) glyceraldehyde-3-phosphate dehydrogenase C sub 1 (GAPC1), a key enzyme in glycolysis. According to PAXdb, some of the above-reported proteins are abundant in the roots.

## Discussion

### Iodine Influences Plant Growth and Development and Can Modulate the Plant Transcriptome

Establishing whether iodine is important for a plant’s life is complex, as it is always present in variable amounts in the soil, water, and atmosphere. Plants can take up iodine from the soil solution through the root system, but they also assimilate it from the air or absorb it through the leaves if dissolved in salt solutions or in rain. All these processes occur naturally (Fuge and Johnson, 1986; Ashworth, 2009), thus a plant cannot be grown in the complete absence of iodine.

To identify whether iodine can act as a micro-nutrient we supplied it to plants at very low concentrations (in the micromolar range). These concentrations are typical of many mineral elements that are beneficial or essential when taken up in low doses, and phytotoxic when in excess (Welch and Shuman, 1995). We observed a difference in plant growth between the control and iodine-treated plants. Where these could be perceived as positive effects of addition of a beneficial compound, these can also be interpreted as a negative effect of removal of iodine from the plant’s nutrition. An increase in biomass and seed production, together with a very evident hastening of flowering, was observed by feeding plants with KIO_3_ (Figure 1 and Supplementary Table S1) or KI (Figure 2) at 0.2 and/or 10 μM. On addition of iodine, flowering was always early and appeared to be specific for iodine, since it was present in the KIO_3_, KI and NaI treatments but completely absent in KBr (Figures 1, 2). However, the positive effects of iodine on growth were lost at 30 μM. This suggests that a concentration of 30 μM applied as I^–^ may be above the toxicity threshold.

In the range of 1–10 μM iodine increases biomass in vegetables, e.g., spinach (Zhu et al., 2003), lettuce (Blasco et al., 2013), tomato (Lehr et al., 1958; Borst Pauwels, 1961), and strawberry (Li et al., 2016), or staple crops, e.g., barley (Borst Pauwels, 1961) and wheat (Cakmak et al., 2017). In tomato, Lehr et al. (1958) demonstrated that treatments with iodine accelerated plant growth, causing early flowering associated with an increase in yield. Similarly, Umaly and Poel (1970) found that the addition of 4 μM KI to the nutrient solution stimulated tomato plants to produce flowers 2–3 days earlier than the control, whereas the use of iodine at a higher concentration (80 μM KI) delayed flower formation and reduced the number of inflorescences. It must be noted that in most biofortification studies with iodine, its native occurrence in nutrient solution or soil of the control plants is not always reported; where iodine concentrations in leaf or root tissue in control plants are reported, these always indicate that iodine was available for uptake and accumulation, regardless of the exogenous administrations (e.g., Borst Pauwels, 1961; Cakmak et al., 2017).

Flowering is a complex physiological process affected by a multitude of internal and external factors, and its hastening may represent an evolutionary adaptive mechanism to guarantee species survival, by optimising the seed set in the case of biotic or abiotic stresses (Ionescu et al., 2017). In Arabidopsis, heat and drought stress are correlated with early flowering, which in turn is generally associated with a reduction in plant growth (Balasubramanian et al., 2006; Schmalenbach et al., 2014). In our study, 10 μM iodate or iodide promoted flowering without negatively impacting on biomass production (Figures 1, 2), which was increased by KIO_3_, thus suggesting the specific flowering-promoting role of iodine in the process.

Our transcriptomic analysis of plants treated with 10 μM KI, NaI, or KBr for 48 h showed that several genes were specifically regulated by iodine, as the large part of DEGs similarly responded to KI and NaI, but not to KBr (Figure 3). This was more evident in root than in shoot tissues, probably because iodine was added to the nutrient solution and was used first by roots before the green parts. Interestingly, transcripts specifically regulated by iodine in the roots were mostly involved in the plant response to biotic and/or abiotic stresses (Figure 4 and Supplementary Tables S6–S8) and the selective regulation of iodine on these groups of genes was also observed in the shoots (Supplementary Tables S4, S5 and Supplementary Figure S4).

Although no previous data are available on the response of Arabidopsis to iodine at the transcriptomic level, the induction of *HALIDE ION METHYLTRANSFERASE*, *SALICYLIC ACID CARBOXYL METHYLTRANSFERASE*, and *SALICYLIC ACID 3-HYDROXYLASE* genes by aromatic iodine compounds, indicating a possible involvement of iodine in the SA metabolism, has already been described in tomato plants (Halka et al., 2019). SA is a signalling molecule involved in local defence reactions at infection sites and the induction of systemic resistance (Vlot et al., 2009).

Iodine likely has an indirect effect on plant resistance given that iodine treatments induce the biosynthesis of several enzymatic or non-enzymatic compounds involved in the plant response to environmental stresses (Leyva et al., 2011; Blasco et al., 2013; Gupta et al., 2015). The antioxidant response induced by increasing KI and KIO_3_ levels was found to be strongly associated with the synthesis of phenolic compounds (Incrocci et al., 2019; Kiferle et al., 2019), in agreement with our transcriptomic data (Figure 4 and Supplementary Table S8). Iodine treatments have also been associated with the modulation of the essential oil composition in basil plants (Kiferle et al., 2019), which plays a key role in defensive and attraction mechanisms in response to the environment (Cseke et al., 2007; Bakkali et al., 2008).

In our study, the relationship between iodine and plant resistance to stress was also suggested by the activation of GO terms associated with hypoxia (Figure 4 and Supplementary Table S8) and the over-representation of data points related to calcium (Ca) regulation (Supplementary Figure S3A). Ca plays a central role in the plant perception of stress by activating a general defence mechanism, which relies on a Ca spiking mechanism and thus on the battery of Ca-dependent proteins that sense Ca and transduce the signal to downstream targets (Dodd et al., 2010; Pucciariello et al., 2012). Our microarray results did not provide evidence that iodine regulated the expression of anaerobic core genes, such as *PYRUVATE DECARBOXYLASE 1* or *ALCOHOL DEHYDROGENASE* (Mustroph et al., 2010). This suggests that the regulation of hypoxic genes by iodine was not associated with limiting O_2_ levels but with an unspecific plant response to an environmental/biotic stress, as it is also highlighted by the activation of enzyme families associated with antioxidant response and xenobiotic detoxification, such as peroxidases, cytochrome P450 or glutathione S-transferases (Supplementary Figure S3B; Mittler et al., 2004; Jouili et al., 2011; Pandian et al., 2020).

At very low doses, iodine thus activates a general defence response which takes place before any biotic or abiotic danger and thus may prepare the plant for a possible future attack or environmentally unfavourable conditions.

The impact of iodine on the transcriptome related to defence was impressive. For evaluation of iodine as a plant nutrient the possible influence on growth and development is also important, and this appeared to be sustained by more scattered elements, requiring further studies. For instance, we found iodine-driven modulation in the shoots of the expression of specific genes that are known to regulate flowering (Xing et al., 2013; Trapalis et al., 2017). This is line with our phenotypical data that indicate that iodine promotes early blooming (Figures 1B, 2A).

### Iodine Is Incorporated Into Plant Proteins

Using radiolabelled iodine, we observed iodine incorporation into leaf and root proteins in various plant species, with a good level of conservation of the molecular mass values (Figure 5), suggesting that iodination plays a functional role in specific proteins. The absence of radioactive bands in maize leaf extracts may be due to a specific characteristic of the species: maize was the only C4 plant analysed in our study.

Under alkaline conditions, iodine is known to react *in vitro* with free amino acids, such as Tyr and His, possibly leading to the formation of several I-labelled proteins (Scott, 1954). In our experiments, however, no radioactive signals were present when ^125^I was added to shoot and root control samples after protein extraction, indicating that protein iodination occurred *in vivo*.

To the best of our knowledge, the presence of naturally occurring iodinated proteins in higher plants has never been described before, although it is well known in seaweed. For instance, the fraction of iodine bound to proteins in *Sargassum kjellanianum* accounts for 65.5% of the total element content of this organism (Hou et al., 2000). In addition, 1D and 2D gel-based electrophoresis combined with laser ablation ICP-MS highlighted several iodinated proteins in Nori seaweed, although no further analyses were conducted to identify them (Romarís-Hortas et al., 2014).

In this study, we identified several iodinated peptides from several high-quality proteomic datasets on plants grown without intentional enrichment of the growing media with iodine, but accumulating the iodine naturally present in soil and water or in MS media. Interestingly, these iodinated peptides belong to proteins, which appeared to be involved in well-defined biological contexts within the plant (Tables 1, 2 and Supplementary Table S10).

In roots, some of the iodinated protein networks that have been identified belong to various class III peroxidases (EC 1.11.17). These are a large molecular family of isozymes present in higher plants, which catalyse redox processes between H_2_O_2_ and reductants and are involved in growth, cell wall differentiation and the response to various biotic/abiotic stresses (Moerschbacher, 1992; Hiraga et al., 2001). They are abundant in root tissues, in which high concentrations of H_2_O_2_ occur during root development (Dunand et al., 2007). In the presence of low concentrations of iodide, various peroxidases catalytically degrade H_2_O_2_ at neutral pH values, generating hypoiodous acid (HIO) (Davies et al., 2008; Vlasova, 2018), a strong iodinating agent that can modify proteins in the environment surrounding the site of its generation, also inducing modification at Tyr. Our findings thus suggest that the class III peroxidases involved in the neutralisation of H_2_O_2_ present in root tissues, in the presence of iodide and resulting HIO, become themselves the targets of iodination, which in our study occurred at different Tyr residues in their structure. In fact we also found that Tyr iodination occurred in other proteins that directly/indirectly interact with or are functionally linked to class III peroxidases, and/or are present at high levels in root tissues (Supplementary Figure S7A and Supplementary Table S11).

Regarding leaves, we identified a number of iodinated peptides from proteins in proteomic datasets from Arabidopsis chloroplast, cauline and rosette extracts (Figure 6B). Most of these proteins are well-known constitutive subunits of molecular complexes (PSII, PSI, Cytb6f and ATPase) present in the plant photosynthetic machinery (Dekker and Boekema, 2005; Nevo et al., 2012). In *in vitro* labelling experiments (Machold and Aurich, 1981) demonstrated that some of them can be iodinated.

The above-mentioned macromolecular assemblies are involved in the generation and transfer of reactive electrons, from their early formation up to the coupling reactions, where their chemical potential allows the generation of plant ATP, NADP and carbohydrates (Foyer, 2018). In this context, high light intensity is one of the major stress factors in green plant tissues, which leads to the production of highly reactive oxygen species (ROS) (^1^O_2_, O_2_^⋅–^, H_2_O_2_), and hydroxyl radicals (Schmitt et al., 2014; Foyer, 2018). These reactions also occur during normal photosynthetic conditions and, whenever not controlled by dedicated endogenous antioxidants, can impact on photosynthetic efficiency.

The principal target of light stress is the chloroplast, which is the preferential site of iodine accumulation in the leaf (Weng et al., 2013), and PSI and PSII are the main sites of O_2_^⋅–^ and ^1^O_2_ production, respectively. High light illumination and the corresponding generation of ROS cause photoinhibition of PSII as well as the modification of other photosynthetic complexes, and induce an accelerated turnover of components of these molecular machineries (Li et al., 2018). The latter phenomenon is generally accomplished through the rapid degradation of photo-damaged proteins and concomitant substitution of them with newly synthetised functional copies (Aro et al., 2005; Yamamoto et al., 2014). This oxidative modification is also known to affect redox-regulated enzymes involved in the Calvin cycle. Proteomic studies have already characterised the nature and the sites of various oxidative and nitrosative modifications at Tyr, Trp and His residues in components of PSII, PSI, Cytb6f and ATP synthase complexes in thylakoid membranes from plants exposed to intense illumination (Galetskiy et al., 2011). These modifications were induced by ROS and/or other reactive nitrogen species, e.g., peroxinitrite, which are formed after the reaction of ROS with nitric oxide and other plant nitrogenous species (Bachi et al., 2013; Lu and Yao, 2018).

Given that such oxidised and nitrated proteins coincide with those found iodinated in our study, ROS likely also react with iodo-containing ions present in the chloroplasts to generate iodinating species that affect Tyr and His residues. We found that iodination processes also affected other proteins functionally related to subunits of PSII, PSI, Cytb6f and ATP synthase complexes, such as those involved in the Calvin Cycle, which are present at high concentrations in the same subcellular district, and have already been reported to directly/indirectly interact with the above-mentioned photosynthetic assemblies (Supplementary Figure S6A and Supplementary Table S11).

The addition of ^1^O_2_ to I^–^ forms peroxyiodide, which decomposes into highly reactive iodine and iodo-containing radicals during dye-mediated photodynamic bacterial inactivation in the presence of potassium iodide (Wen et al., 2017). This process is fundamental for antimicrobial photodynamic therapy (Hamblin and Abrahamse, 2018). Thus, in the presence of iodide, the above-mentioned photoactivated reactions or their possible process variants may have contributed to generate highly reactive iodo-containing molecules and/or radicals, thereby leading to the formation of the iodinated proteins observed in this study. The possible functional meaning of these is certainly worth studying.

## Conclusion

We demonstrated that very low amounts of iodine (between 0.20 and 10 μM, i.e., in the range of the concentrations required by plants for several micro-nutrients) improved plant growth and development thus promoting both biomass production and early flowering, and that this effect could not be achieved by another halogen most resembling iodine (Br). Secondly, we found that iodine was able to modulate gene expression in a specific way, activating multiple pathways, mostly involved in defence responses. Finally, we demonstrated that iodine can be a structural component of several different proteins, and conserved iodinated proteins are synthesised in both the roots and shoots of phylogenetically distant species.

These three lines of evidence highlight that iodine has a nutritional role in plants. This means that the influence of iodine on plants is not merely the result of an indirect priming effect by a potentially phytotoxic compound. Considering that plant nutrients are chemical elements that are components of biological molecules and/or influence essential metabolic functions, iodine matches at least the first part of this definition. Further studies on the importance of organification of iodine in proteins on their catalytic and/or regulatory function will help to complete the picture on the functional role of iodine as a plant nutrient.

## Data Availability Statement

The datasets presented in this study can be found in online repositories. The names of the repository/repositories and accession number(s) can be found below: https://www.ncbi.nlm.nih.gov/geo/, GSE157643.

## Author Contributions

PP, CK, MM, KH, and HTH: conceived the project. CK, MM, and SB: experiments on plant phenotype and transcriptomic. PS, CK, and MM: experiments on radioactive feeding. AMS: bioinformatics analysis of mass spectrometry-based proteomics data sets. CK and AMS: data analysis and figures preparation. CK, AMS, AS, and SG: writing—original draft preparation. PP, AS, SG, PS, KH, and HTH: general discussion and revision of the article. All authors read and contributed to previous versions and approved the final version.

## Conflict of Interest

KH and HH were employees of SQM International N.V., a company active in the sector of fertilisers. The remaining authors declare that the research was conducted in the absence of any commercial or financial relationships that could be construed as a potential conflict of interest.

## Footnotes

1 http://cbl-gorilla.cs.technion.ac.il

2 http://mapman.gabipd.org/mapman

3 https://genevestigator.com

4 http://medgen.ugent.be/j̃vdesomp/genorm

5 https://www.ebi.ac.uk/pride/archive

6 www.arabidopsis.org

7 https://www.maxquant.org/maxquant/

8 http://mapman.gabipd.org/web/guest/app/Mercator

9 http://string-db.org
