# Supplementary material for: Evidences for a Nutritional Role of Iodine in Plants
Source: Front Plant Sci. 2021 Feb 17;12:616868. doi: 10.3389/fpls.2021.616868 (PMC7925997; doi:10.3389/fpls.2021.616868)
Supplement: Supplementary file 9 [file Table_1.DOCX]

**Table S1:** Primers used for RT-qPCR gene expression analysis.

| **Gene (AGI)** | **Forward primer** | **Reverse primer** |
| --- | --- | --- |
| At4g05320 (UBQ10) | GGCCTTGTATAATCCCTGATGAATAAG | AAAGAGATAACAGGAACGGAAACATAGT |
| At2g25810.1 (TIP4) | TGATCTTCCCAATGGCTAAGG | TGATCTCCCAATGGCTAAGG |
| At5g52390 | GGTTAAATGCTTAAACCAATGTCC | CTTCTTGACTTCCCTTCTTGTGAT |
| At3g57260 | GCTTCCTTCTTCAACCACACAGC | TGGCAAGGTATCGCCTAGCATC |
| At1g56600 | AAGAAGCAACAGACACTTCAGCAG | TGAAGAGGCGTATGCAGCAAC |
| At1g75750 | TTCTCCAACTCGTCCAGGCTGA | TACACACGCACTCCCACAATCG |
| At4g15680 | GATAGAGCAAGCATTGGCTCAG | GAACCAGAGAGCGATTGAGATG |
| At2g36885 | GGAGCAACTGGTGGTGTTATATCC | CTGTAACAGCGTGCGGACTTTC |
| At5g13320 | GTTGTCACAAATTTCGCTGGCTTG | GCGCGTTGTTGTAGAAACCAGTC |
| At1g75040 | ATCACCCACAGCACAGAGACAC | AGCAATGCCGCTTGTGATGAAC |
| At4g23680 | ACCATGTCTTCCCTGATGCTATCG | TGAACACCTCCTCCTTTCCATCCC |
| At1g34510 | CCGAACGGTTACTGCAGCATTG | GGAAGCATCACAACCTTTGACAAC |
| At4g25220 | TACGTCAGCCACAACATGATCGG | CGACAAGTTTCCTGATGTCTCCTC |
| At1g13080 | TTGATGATCACTTGAAGCCAGAGG | CCCGCGAGAAATACATCCATGAC |
