# Supplementary material for: Evidences for a Nutritional Role of Iodine in Plants
Source: Front Plant Sci. 2021 Feb 17;12:616868. doi: 10.3389/fpls.2021.616868 (PMC7925997; doi:10.3389/fpls.2021.616868)
Supplement: Supplementary file 11 [file Table_3.docx]

**Table S3.** Effect of different KIO_3_ concentrations (0, 0.20 and 10 μM) in the nutrient solution on rosette and inflorescence FW and DW, seed production and number of produced siliques/plant. Each value is the mean (± standard error, SE) of 45 biological replicates, consisting of a single plant, with the exception of data on seed production: the cultivation system did not allow to harvest seeds from single plants, therefore the average of produced seed/tray (3 different trays; 15 plants/tray) was compared and statistic analysis was performed accordingly. Values indicated by different superscript letters significantly differ from each other (according with one-way ANOVA, LSD posthoc test, P ≤ 0.05).

|  | **Control** | **KIO_3_ 0.20 µM** | **KIO_3_ 10 µM** | **Significance** |
| --- | --- | --- | --- | --- |
| Rosette FW (g) | 1.22 ± 0.040 | 1.19 ± 0.033 | 1.27 ± 0.033 | n.s. |
| Inflorescence FW (g) | 0.25 ± 0.031 ^b^ | 0.39 ± 0.038 ^a^ | 0.39 ± 0.032 ^a^ | *P-value* ≤0.005 |
| Rosette DW (g) | 0.11 ± 0.004 | 0.11 ± 0.004 | 0.12 ± 0.004 | n.s. |
| Inflorescence DW (g) | 0.03 ± 0.003 ^b^ | 0.05 ± 0.004 ^a^ | 0.04 ± 0.004 ^a^ | *P-value* ≤0.005 |
| Seed production* (g) | 7.00 ± 1.130 | 10.50 ± 1.751 | 9.47 ± 2.062 | n.s. |
| Seed/silique (n°) | 58.59 ± 1.184 | 59.09 ± 1.329 | 59.70 ± 0.740 | n.s. |

* Seed production per hydroponic tray, consisting of 15 plants.
