# Supplementary material for: Evidences for a Nutritional Role of Iodine in Plants
Source: Front Plant Sci. 2021 Feb 17;12:616868. doi: 10.3389/fpls.2021.616868 (PMC7925997; doi:10.3389/fpls.2021.616868)
Supplement: Supplementary file 16 [file Table_8.DOCX]

**Table S8.** List of the main biological processes affected by iodine based on the GO terms enrichment analysis in root tissues (only genes regulated in NaI- and KI-treated plants, and not in KBr-treated plants, when compared with the control were analyzed). Data were extracted from Gorilla (http://cbl-gorilla.cs.technion.ac.il). In this analysis, DEGs with log2FC≥2.5 or log2FC≤-2.5 were used. 'P-value' is the enrichment p-value computed according to the mHG or HG model. This p-value is not corrected for multiple testing of 4037 GO terms. 'FDR q-value' is the correction of the above p-value for multiple testing using the Benjamini and Hochberg (1995) method (BH procedure). Namely, for the i^th^ term (ranked according to p-value) the FDR q-value is (p-value * number of GO terms) / i. Enrichment (N, B, n, b) is defined as follows: N - is the total number of genes; B - is the total number of genes associated with a specific GO term; n - is the number of genes in the top of the user's input list or in the target set when appropriate; b - is the number of genes in the intersection. Enrichment = (b/n) / (B/N). The genes classified in each GO term are also listed.

| **GO term** | **Description** | [**P-value**](http://cbl-gorilla.cs.technion.ac.il/GOrilla/akje8mfg/GOResultsPROCESS.html#p_value_info) | [**FDR q-value**](http://cbl-gorilla.cs.technion.ac.il/GOrilla/akje8mfg/GOResultsPROCESS.html#fdr_info) | [**Enrichment (N, B, n, b)**](http://cbl-gorilla.cs.technion.ac.il/GOrilla/akje8mfg/GOResultsPROCESS.html#enrich_info) | [**Genes**](http://cbl-gorilla.cs.technion.ac.il/GOrilla/akje8mfg/GOResultsPROCESS.html#genes_info) |
| --- | --- | --- | --- | --- | --- |
| [GO:0050896](http://www.godatabase.org/cgi-bin/amigo/go.cgi?query=GO:0050896&view=details) | response to stimulus | 1.25E-11 | 5.03E-8 | 2.77 (11861,1303,161,49) | [[-] Hide genes](javascript:toggle('elements_GO:0050896'))  AT5G22530 - hypothetical protein AT5G18470 - curculin-like (mannose-binding) lectin family protein AT4G37290 - hypothetical protein AT2G21210 - saur-like auxin-responsive protein AT4G28460 - hypothetical protein AT1G66090 - tir-nbs class of disease resistance protein AT1G68620 - probable carboxylesterase 6 AT1G15010 - hypothetical protein AT1G57630 - toll-interleukin-resistance domain-containing protein AT4G36430 - peroxidase 49 AT1G12200 - putative flavin monooxygenase. AT5G65600 - concanavalin a-like lectin kinase-like protein AT1G35910 - probable trehalose-phosphate phosphatase d AT3G23170 - hypothetical protein AT3G16530 - legume lectin-like protein AT1G19020 - hypothetical protein AT5G06730 - peroxidase AT1G73805 - protein sar deficient 1 AT4G10500 - oxidoreductase, 2og-fe(ii) oxygenase family protein AT5G05340 - peroxidase 52 AT4G37710 - vq motif-containing protein AT5G19880 - peroxidase AT2G22880 - vq motif-containing protein AT1G26380 - fad-binding and bbe domain-containing protein AT1G34510 - peroxidase 8 AT1G72910 - toll-interleukin-resistance domain-containing protein AT1G26410 - fad-binding and bbe domain-containing protein AT1G72940 - toll-interleukin-resistance domain-containing protein AT1G14550 - peroxidase 5 AT3G21670 - nitrate transporter 1.3 AT1G14540 - peroxidase 4 AT5G39580 - peroxidase 62 AT4G08780 - peroxidase 38 AT4G23680 - polyketide cyclase/dehydrase and lipid transport superfamily protein AT1G65500 - hypothetical protein AT3G45710 - major facilitator superfamily protein AT5G10760 - aspartyl protease family protein AT3G28580 - aaa-type atpase family protein AT2G38340 - dehydration-responsive element-binding protein 2e AT3G55790 - hypothetical protein AT1G43910 - p-loop containing nucleoside triphosphate hydrolases superfamily protein AT5G22270 - hypothetical protein AT2G41380 - s-adenosyl-l-methionine-dependent methyltransferase-like protein AT5G38710 - proline dehydrogenase 2 AT2G29250 - concanavalin a-like lectin protein kinase-like protein AT2G29220 - putative inactive l-type lectin-domain containing receptor kinase iii.1 AT4G37220 - cold acclimation protein wcor413 AT5G38900 - thioredoxin superfamily protein AT1G70880 - srpbcc domain-containing protein |
| [GO:0006950](http://www.godatabase.org/cgi-bin/amigo/go.cgi?query=GO:0006950&view=details) | response to stress | 3.23E-11 | 6.53E-8 | 3.23 (11861,890,161,39) | [[-] Hide genes](javascript:toggle('elements_GO:0006950'))  AT4G08780 - peroxidase 38 AT5G22530 - hypothetical protein AT4G23680 - polyketide cyclase/dehydrase and lipid transport superfamily protein AT1G65500 - hypothetical protein AT4G28460 - hypothetical protein AT5G10760 - aspartyl protease family protein AT1G66090 - tir-nbs class of disease resistance protein AT1G68620 - probable carboxylesterase 6 AT1G57630 - toll-interleukin-resistance domain-containing protein AT1G15010 - hypothetical protein AT3G28580 - aaa-type atpase family protein AT4G36430 - peroxidase 49 AT1G12200 - putative flavin monooxygenase. AT5G65600 - concanavalin a-like lectin kinase-like protein AT1G35910 - probable trehalose-phosphate phosphatase d AT2G38340 - dehydration-responsive element-binding protein 2e AT3G55790 - hypothetical protein AT3G23170 - hypothetical protein AT1G19020 - hypothetical protein AT5G06730 - peroxidase AT5G22270 - hypothetical protein AT1G73805 - protein sar deficient 1 AT4G10500 - oxidoreductase, 2og-fe(ii) oxygenase family protein AT5G19880 - peroxidase AT4G37710 - vq motif-containing protein AT5G05340 - peroxidase 52 AT5G38710 - proline dehydrogenase 2 AT1G26380 - fad-binding and bbe domain-containing protein AT1G72910 - toll-interleukin-resistance domain-containing protein AT1G34510 - peroxidase 8 AT2G29250 - concanavalin a-like lectin protein kinase-like protein AT2G29220 - putative inactive l-type lectin-domain containing receptor kinase iii.1 AT1G26410 - fad-binding and bbe domain-containing protein AT1G72940 - toll-interleukin-resistance domain-containing protein AT1G14550 - peroxidase 5 AT5G38900 - thioredoxin superfamily protein AT1G14540 - peroxidase 4 AT5G39580 - peroxidase 62 AT1G70880 - srpbcc domain-containing protein |
| [GO:0071453](http://www.godatabase.org/cgi-bin/amigo/go.cgi?query=GO:0071453&view=details) | cellular response to oxygen levels | 2.15E-10 | 2.89E-7 | 9.46 (11861,109,161,14) | [[-] Hide genes](javascript:toggle('elements_GO:0071453'))  AT1G19020 - hypothetical protein AT1G66090 - tir-nbs class of disease resistance protein AT4G37710 - vq motif-containing protein AT1G68620 - probable carboxylesterase 6 AT1G57630 - toll-interleukin-resistance domain-containing protein AT1G15010 - hypothetical protein AT1G26380 - fad-binding and bbe domain-containing protein AT1G72910 - toll-interleukin-resistance domain-containing protein AT1G26410 - fad-binding and bbe domain-containing protein AT1G72940 - toll-interleukin-resistance domain-containing protein AT1G14550 - peroxidase 5 AT1G14540 - peroxidase 4 AT3G55790 - hypothetical protein AT3G23170 - hypothetical protein |
| [GO:0071456](http://www.godatabase.org/cgi-bin/amigo/go.cgi?query=GO:0071456&view=details) | cellular response to hypoxia | 2.15E-10 | 2.17E-7 | 9.46 (11861,109,161,14) | [[-] Hide genes](javascript:toggle('elements_GO:0071456'))  AT1G19020 - hypothetical protein AT1G66090 - tir-nbs class of disease resistance protein AT4G37710 - vq motif-containing protein AT1G68620 - probable carboxylesterase 6 AT1G57630 - toll-interleukin-resistance domain-containing protein AT1G15010 - hypothetical protein AT1G26380 - fad-binding and bbe domain-containing protein AT1G72910 - toll-interleukin-resistance domain-containing protein AT1G26410 - fad-binding and bbe domain-containing protein AT1G72940 - toll-interleukin-resistance domain-containing protein AT1G14550 - peroxidase 5 AT1G14540 - peroxidase 4 AT3G55790 - hypothetical protein AT3G23170 - hypothetical protein |
| [GO:0036294](http://www.godatabase.org/cgi-bin/amigo/go.cgi?query=GO:0036294&view=details) | cellular response to decreased oxygen levels | 2.15E-10 | 1.74E-7 | 9.46 (11861,109,161,14) | [[-] Hide genes](javascript:toggle('elements_GO:0036294'))  AT1G19020 - hypothetical protein AT1G66090 - tir-nbs class of disease resistance protein AT4G37710 - vq motif-containing protein AT1G68620 - probable carboxylesterase 6 AT1G57630 - toll-interleukin-resistance domain-containing protein AT1G15010 - hypothetical protein AT1G26380 - fad-binding and bbe domain-containing protein AT1G72910 - toll-interleukin-resistance domain-containing protein AT1G26410 - fad-binding and bbe domain-containing protein AT1G72940 - toll-interleukin-resistance domain-containing protein AT1G14550 - peroxidase 5 AT1G14540 - peroxidase 4 AT3G55790 - hypothetical protein AT3G23170 - hypothetical protein |
| [GO:0001666](http://www.godatabase.org/cgi-bin/amigo/go.cgi?query=GO:0001666&view=details) | response to hypoxia | 2.75E-10 | 1.85E-7 | 9.29 (11861,111,161,14) | [[-] Hide genes](javascript:toggle('elements_GO:0001666'))  AT1G19020 - hypothetical protein AT1G66090 - tir-nbs class of disease resistance protein AT4G37710 - vq motif-containing protein AT1G68620 - probable carboxylesterase 6 AT1G57630 - toll-interleukin-resistance domain-containing protein AT1G15010 - hypothetical protein AT1G26380 - fad-binding and bbe domain-containing protein AT1G72910 - toll-interleukin-resistance domain-containing protein AT1G26410 - fad-binding and bbe domain-containing protein AT1G72940 - toll-interleukin-resistance domain-containing protein AT1G14550 - peroxidase 5 AT1G14540 - peroxidase 4 AT3G55790 - hypothetical protein AT3G23170 - hypothetical protein |
| [GO:0036293](http://www.godatabase.org/cgi-bin/amigo/go.cgi?query=GO:0036293&view=details) | response to decreased oxygen levels | 3.11E-10 | 1.79E-7 | 9.21 (11861,112,161,14) | [[-] Hide genes](javascript:toggle('elements_GO:0036293'))  AT1G19020 - hypothetical protein AT1G66090 - tir-nbs class of disease resistance protein AT4G37710 - vq motif-containing protein AT1G68620 - probable carboxylesterase 6 AT1G57630 - toll-interleukin-resistance domain-containing protein AT1G15010 - hypothetical protein AT1G26380 - fad-binding and bbe domain-containing protein AT1G72910 - toll-interleukin-resistance domain-containing protein AT1G26410 - fad-binding and bbe domain-containing protein AT1G72940 - toll-interleukin-resistance domain-containing protein AT1G14550 - peroxidase 5 AT1G14540 - peroxidase 4 AT3G55790 - hypothetical protein AT3G23170 - hypothetical protein |
| [GO:0070482](http://www.godatabase.org/cgi-bin/amigo/go.cgi?query=GO:0070482&view=details) | response to oxygen levels | 3.51E-10 | 1.77E-7 | 9.13 (11861,113,161,14) | [[-] Hide genes](javascript:toggle('elements_GO:0070482'))  AT1G19020 - hypothetical protein AT1G66090 - tir-nbs class of disease resistance protein AT4G37710 - vq motif-containing protein AT1G68620 - probable carboxylesterase 6 AT1G57630 - toll-interleukin-resistance domain-containing protein AT1G15010 - hypothetical protein AT1G26380 - fad-binding and bbe domain-containing protein AT1G72910 - toll-interleukin-resistance domain-containing protein AT1G26410 - fad-binding and bbe domain-containing protein AT1G72940 - toll-interleukin-resistance domain-containing protein AT1G14550 - peroxidase 5 AT1G14540 - peroxidase 4 AT3G55790 - hypothetical protein AT3G23170 - hypothetical protein |
| [GO:0042221](http://www.godatabase.org/cgi-bin/amigo/go.cgi?query=GO:0042221&view=details) | response to chemical | 3.43E-9 | 1.54E-6 | 3.58 (11861,577,161,28) | [[-] Hide genes](javascript:toggle('elements_GO:0042221'))  AT5G22530 - hypothetical protein AT2G21210 - saur-like auxin-responsive protein AT1G65500 - hypothetical protein AT3G45710 - major facilitator superfamily protein AT1G66090 - tir-nbs class of disease resistance protein AT1G68620 - probable carboxylesterase 6 AT1G57630 - toll-interleukin-resistance domain-containing protein AT1G15010 - hypothetical protein AT3G28580 - aaa-type atpase family protein AT2G38340 - dehydration-responsive element-binding protein 2e AT3G55790 - hypothetical protein AT3G23170 - hypothetical protein AT3G16530 - legume lectin-like protein AT1G43910 - p-loop containing nucleoside triphosphate hydrolases superfamily protein AT1G19020 - hypothetical protein AT1G73805 - protein sar deficient 1 AT2G41380 - s-adenosyl-l-methionine-dependent methyltransferase-like protein AT4G10500 - oxidoreductase, 2og-fe(ii) oxygenase family protein AT4G37710 - vq motif-containing protein AT5G38710 - proline dehydrogenase 2 AT1G26380 - fad-binding and bbe domain-containing protein AT1G72910 - toll-interleukin-resistance domain-containing protein AT1G26410 - fad-binding and bbe domain-containing protein AT1G72940 - toll-interleukin-resistance domain-containing protein AT3G21670 - nitrate transporter 1.3 AT1G14550 - peroxidase 5 AT4G37220 - cold acclimation protein wcor413 AT1G14540 - peroxidase 4 |
| [GO:0070887](http://www.godatabase.org/cgi-bin/amigo/go.cgi?query=GO:0070887&view=details) | cellular response to chemical stimulus | 1.04E-8 | 4.2E-6 | 6.46 (11861,171,161,15) | [[-] Hide genes](javascript:toggle('elements_GO:0070887'))  AT1G19020 - hypothetical protein AT3G45710 - major facilitator superfamily protein AT1G66090 - tir-nbs class of disease resistance protein AT4G37710 - vq motif-containing protein AT1G68620 - probable carboxylesterase 6 AT1G57630 - toll-interleukin-resistance domain-containing protein AT1G15010 - hypothetical protein AT1G26380 - fad-binding and bbe domain-containing protein AT1G72910 - toll-interleukin-resistance domain-containing protein AT1G26410 - fad-binding and bbe domain-containing protein AT1G72940 - toll-interleukin-resistance domain-containing protein AT1G14550 - peroxidase 5 AT1G14540 - peroxidase 4 AT3G55790 - hypothetical protein AT3G23170 - hypothetical protein |
| [GO:0009628](http://www.godatabase.org/cgi-bin/amigo/go.cgi?query=GO:0009628&view=details) | response to abiotic stimulus | 3.07E-7 | 1.13E-4 | 3.36 (11861,504,161,23) | [[-] Hide genes](javascript:toggle('elements_GO:0009628'))  AT1G19020 - hypothetical protein AT1G73805 - protein sar deficient 1 AT5G22270 - hypothetical protein AT5G18470 - curculin-like (mannose-binding) lectin family protein AT4G37290 - hypothetical protein AT1G65500 - hypothetical protein AT4G37710 - vq motif-containing protein AT1G66090 - tir-nbs class of disease resistance protein AT1G68620 - probable carboxylesterase 6 AT1G15010 - hypothetical protein AT1G57630 - toll-interleukin-resistance domain-containing protein AT2G22880 - vq motif-containing protein AT5G38710 - proline dehydrogenase 2 AT1G26380 - fad-binding and bbe domain-containing protein AT1G72910 - toll-interleukin-resistance domain-containing protein AT1G26410 - fad-binding and bbe domain-containing protein AT1G72940 - toll-interleukin-resistance domain-containing protein AT1G35910 - probable trehalose-phosphate phosphatase d AT2G38340 - dehydration-responsive element-binding protein 2e AT1G14550 - peroxidase 5 AT3G55790 - hypothetical protein AT1G14540 - peroxidase 4 AT3G23170 - hypothetical protein |
| [GO:0051704](http://www.godatabase.org/cgi-bin/amigo/go.cgi?query=GO:0051704&view=details) | multi-organism process | 5.26E-7 | 1.77E-4 | 4.80 (11861,230,161,15) | [[-] Hide genes](javascript:toggle('elements_GO:0051704'))  AT1G73805 - protein sar deficient 1 AT4G28460 - hypothetical protein AT4G10500 - oxidoreductase, 2og-fe(ii) oxygenase family protein AT5G10760 - aspartyl protease family protein AT1G15010 - hypothetical protein AT1G23160 - auxin-responsive gh3 family protein AT2G29250 - concanavalin a-like lectin protein kinase-like protein AT4G36430 - peroxidase 49 AT2G29220 - putative inactive l-type lectin-domain containing receptor kinase iii.1 AT1G12200 - putative flavin monooxygenase. AT5G65600 - concanavalin a-like lectin kinase-like protein AT5G39580 - peroxidase 62 AT5G38900 - thioredoxin superfamily protein AT3G23170 - hypothetical protein AT3G16530 - legume lectin-like protein |
| [GO:0006979](http://www.godatabase.org/cgi-bin/amigo/go.cgi?query=GO:0006979&view=details) | response to oxidative stress | 1.35E-6 | 4.2E-4 | 5.67 (11861,156,161,12) | [[-] Hide genes](javascript:toggle('elements_GO:0006979'))  AT4G08780 - peroxidase 38 AT1G19020 - hypothetical protein AT1G34510 - peroxidase 8 AT5G06730 - peroxidase AT3G28580 - aaa-type atpase family protein AT4G36430 - peroxidase 49 AT5G05340 - peroxidase 52 AT5G19880 - peroxidase AT1G35910 - probable trehalose-phosphate phosphatase d AT1G14550 - peroxidase 5 AT1G14540 - peroxidase 4 AT5G39580 - peroxidase 62 |
| [GO:0006952](http://www.godatabase.org/cgi-bin/amigo/go.cgi?query=GO:0006952&view=details) | defense response | 1.67E-6 | 4.81E-4 | 4.39 (11861,252,161,15) | [[-] Hide genes](javascript:toggle('elements_GO:0006952'))  AT4G23680 - polyketide cyclase/dehydrase and lipid transport superfamily protein AT1G73805 - protein sar deficient 1 AT4G28460 - hypothetical protein AT4G10500 - oxidoreductase, 2og-fe(ii) oxygenase family protein AT1G66090 - tir-nbs class of disease resistance protein AT5G10760 - aspartyl protease family protein AT1G15010 - hypothetical protein AT2G29250 - concanavalin a-like lectin protein kinase-like protein AT2G29220 - putative inactive l-type lectin-domain containing receptor kinase iii.1 AT1G12200 - putative flavin monooxygenase. AT5G65600 - concanavalin a-like lectin kinase-like protein AT5G39580 - peroxidase 62 AT5G38900 - thioredoxin superfamily protein AT1G70880 - srpbcc domain-containing protein AT3G23170 - hypothetical protein |
| [GO:0051707](http://www.godatabase.org/cgi-bin/amigo/go.cgi?query=GO:0051707&view=details) | response to other organism | 1.91E-6 | 5.14E-4 | 4.65 (11861,222,161,14) | [[-] Hide genes](javascript:toggle('elements_GO:0051707'))  AT1G73805 - protein sar deficient 1 AT4G28460 - hypothetical protein AT4G10500 - oxidoreductase, 2og-fe(ii) oxygenase family protein AT5G10760 - aspartyl protease family protein AT1G15010 - hypothetical protein AT2G29250 - concanavalin a-like lectin protein kinase-like protein AT4G36430 - peroxidase 49 AT2G29220 - putative inactive l-type lectin-domain containing receptor kinase iii.1 AT1G12200 - putative flavin monooxygenase. AT5G65600 - concanavalin a-like lectin kinase-like protein AT5G39580 - peroxidase 62 AT5G38900 - thioredoxin superfamily protein AT3G23170 - hypothetical protein AT3G16530 - legume lectin-like protein |
| [GO:0043207](http://www.godatabase.org/cgi-bin/amigo/go.cgi?query=GO:0043207&view=details) | response to external biotic stimulus | 2.48E-6 | 6.26E-4 | 4.54 (11861,227,161,14) | [[-] Hide genes](javascript:toggle('elements_GO:0043207'))  AT1G73805 - protein sar deficient 1 AT4G28460 - hypothetical protein AT4G10500 - oxidoreductase, 2og-fe(ii) oxygenase family protein AT5G10760 - aspartyl protease family protein AT1G15010 - hypothetical protein AT2G29250 - concanavalin a-like lectin protein kinase-like protein AT4G36430 - peroxidase 49 AT2G29220 - putative inactive l-type lectin-domain containing receptor kinase iii.1 AT1G12200 - putative flavin monooxygenase. AT5G65600 - concanavalin a-like lectin kinase-like protein AT5G39580 - peroxidase 62 AT5G38900 - thioredoxin superfamily protein AT3G23170 - hypothetical protein AT3G16530 - legume lectin-like protein |
| [GO:0009607](http://www.godatabase.org/cgi-bin/amigo/go.cgi?query=GO:0009607&view=details) | response to biotic stimulus | 2.48E-6 | 5.9E-4 | 4.54 (11861,227,161,14) | [[-] Hide genes](javascript:toggle('elements_GO:0009607'))  AT1G73805 - protein sar deficient 1 AT4G28460 - hypothetical protein AT4G10500 - oxidoreductase, 2og-fe(ii) oxygenase family protein AT5G10760 - aspartyl protease family protein AT1G15010 - hypothetical protein AT2G29250 - concanavalin a-like lectin protein kinase-like protein AT4G36430 - peroxidase 49 AT2G29220 - putative inactive l-type lectin-domain containing receptor kinase iii.1 AT1G12200 - putative flavin monooxygenase. AT5G65600 - concanavalin a-like lectin kinase-like protein AT5G39580 - peroxidase 62 AT5G38900 - thioredoxin superfamily protein AT3G23170 - hypothetical protein AT3G16530 - legume lectin-like protein |
| [GO:0098542](http://www.godatabase.org/cgi-bin/amigo/go.cgi?query=GO:0098542&view=details) | defense response to other organism | 4.51E-6 | 1.01E-3 | 5.05 (11861,175,161,12) | [[-] Hide genes](javascript:toggle('elements_GO:0098542'))  AT2G29250 - concanavalin a-like lectin protein kinase-like protein AT1G73805 - protein sar deficient 1 AT2G29220 - putative inactive l-type lectin-domain containing receptor kinase iii.1 AT1G12200 - putative flavin monooxygenase. AT4G10500 - oxidoreductase, 2og-fe(ii) oxygenase family protein AT5G65600 - concanavalin a-like lectin kinase-like protein AT4G28460 - hypothetical protein AT5G10760 - aspartyl protease family protein AT1G15010 - hypothetical protein AT5G38900 - thioredoxin superfamily protein AT5G39580 - peroxidase 62 AT3G23170 - hypothetical protein |
| [GO:0051716](http://www.godatabase.org/cgi-bin/amigo/go.cgi?query=GO:0051716&view=details) | cellular response to stimulus | 7.74E-6 | 1.64E-3 | 3.48 (11861,360,161,17) | [[-] Hide genes](javascript:toggle('elements_GO:0051716'))  AT1G19020 - hypothetical protein AT1G73805 - protein sar deficient 1 AT3G45710 - major facilitator superfamily protein AT1G66090 - tir-nbs class of disease resistance protein AT4G37710 - vq motif-containing protein AT1G68620 - probable carboxylesterase 6 AT1G15010 - hypothetical protein AT1G57630 - toll-interleukin-resistance domain-containing protein AT1G26380 - fad-binding and bbe domain-containing protein AT1G72910 - toll-interleukin-resistance domain-containing protein AT1G26410 - fad-binding and bbe domain-containing protein AT1G72940 - toll-interleukin-resistance domain-containing protein AT2G38340 - dehydration-responsive element-binding protein 2e AT1G14550 - peroxidase 5 AT3G55790 - hypothetical protein AT1G14540 - peroxidase 4 AT3G23170 - hypothetical protein |
| [GO:0009605](http://www.godatabase.org/cgi-bin/amigo/go.cgi?query=GO:0009605&view=details) | response to external stimulus | 2.47E-5 | 4.99E-3 | 3.51 (11861,315,161,15) | [[-] Hide genes](javascript:toggle('elements_GO:0009605'))  AT1G73805 - protein sar deficient 1 AT4G28460 - hypothetical protein AT4G10500 - oxidoreductase, 2og-fe(ii) oxygenase family protein AT5G10760 - aspartyl protease family protein AT1G15010 - hypothetical protein AT3G28580 - aaa-type atpase family protein AT2G29250 - concanavalin a-like lectin protein kinase-like protein AT4G36430 - peroxidase 49 AT2G29220 - putative inactive l-type lectin-domain containing receptor kinase iii.1 AT1G12200 - putative flavin monooxygenase. AT5G65600 - concanavalin a-like lectin kinase-like protein AT5G39580 - peroxidase 62 AT5G38900 - thioredoxin superfamily protein AT3G23170 - hypothetical protein AT3G16530 - legume lectin-like protein |
| [GO:0033554](http://www.godatabase.org/cgi-bin/amigo/go.cgi?query=GO:0033554&view=details) | cellular response to stress | 2.97E-5 | 5.71E-3 | 3.45 (11861,320,161,15) | [[-] Hide genes](javascript:toggle('elements_GO:0033554'))  AT1G19020 - hypothetical protein AT1G66090 - tir-nbs class of disease resistance protein AT4G37710 - vq motif-containing protein AT1G68620 - probable carboxylesterase 6 AT1G15010 - hypothetical protein AT1G57630 - toll-interleukin-resistance domain-containing protein AT1G26380 - fad-binding and bbe domain-containing protein AT1G72910 - toll-interleukin-resistance domain-containing protein AT1G26410 - fad-binding and bbe domain-containing protein AT1G72940 - toll-interleukin-resistance domain-containing protein AT2G38340 - dehydration-responsive element-binding protein 2e AT1G14550 - peroxidase 5 AT3G55790 - hypothetical protein AT1G14540 - peroxidase 4 AT3G23170 - hypothetical protein |
| [GO:0018958](http://www.godatabase.org/cgi-bin/amigo/go.cgi?query=GO:0018958&view=details) | phenol-containing compound metabolic process | 4.76E-5 | 8.74E-3 | 36.84 (11861,6,161,3) | [[-] Hide genes](javascript:toggle('elements_GO:0018958'))  AT1G64160 - dirigent-like protein dir5 AT4G10500 - oxidoreductase, 2og-fe(ii) oxygenase family protein AT5G63560 - hxxxd-type acyl-transferase-like protein |
| [GO:0002239](http://www.godatabase.org/cgi-bin/amigo/go.cgi?query=GO:0002239&view=details) | response to oomycetes | 5.53E-5 | 9.7E-3 | 8.84 (11861,50,161,6) | [[-] Hide genes](javascript:toggle('elements_GO:0002239'))  AT2G29250 - concanavalin a-like lectin protein kinase-like protein AT1G73805 - protein sar deficient 1 AT2G29220 - putative inactive l-type lectin-domain containing receptor kinase iii.1 AT4G10500 - oxidoreductase, 2og-fe(ii) oxygenase family protein AT5G65600 - concanavalin a-like lectin kinase-like protein AT3G16530 - legume lectin-like protein |
| [GO:0002229](http://www.godatabase.org/cgi-bin/amigo/go.cgi?query=GO:0002229&view=details) | defense response to oomycetes | 1.94E-4 | 3.26E-2 | 9.21 (11861,40,161,5) | [[-] Hide genes](javascript:toggle('elements_GO:0002229'))  AT2G29250 - concanavalin a-like lectin protein kinase-like protein AT1G73805 - protein sar deficient 1 AT2G29220 - putative inactive l-type lectin-domain containing receptor kinase iii.1 AT4G10500 - oxidoreductase, 2og-fe(ii) oxygenase family protein AT5G65600 - concanavalin a-like lectin kinase-like protein |
| [GO:0046189](http://www.godatabase.org/cgi-bin/amigo/go.cgi?query=GO:0046189&view=details) | phenol-containing compound biosynthetic process | 5.44E-4 | 8.79E-2 | 49.11 (11861,3,161,2) | [[-] Hide genes](javascript:toggle('elements_GO:0046189'))  AT1G64160 - dirigent-like protein dir5 AT5G63560 - hxxxd-type acyl-transferase-like protein |
