# Supplementary material for: Evidences for a Nutritional Role of Iodine in Plants
Source: Front Plant Sci. 2021 Feb 17;12:616868. doi: 10.3389/fpls.2021.616868 (PMC7925997; doi:10.3389/fpls.2021.616868)
Supplement: Supplementary file 17 [file Table_9.DOCX]

**Table S9.** List of the molecular functions affected by iodine based on the GO terms enrichment analysis in root tissues (only genes regulated in NaI- and KI-treated plants, and not in KBr-treated plants, when compared with the control were analyzed). Data were extracted from Gorilla (http://cbl-gorilla.cs.technion.ac.il). In this analysis, DEGs with log2FC≥2.5 or log2FC≤-2.5 were used. 'P-value' is the enrichment p-value computed according to the mHG or HG model. This p-value is not corrected for multiple testing of 1737 GO terms. 'FDR q-value' is the correction of the above p-value for multiple testing using the Benjamini and Hochberg (1995) method (BH procedure). Namely, for the i^th^ term (ranked according to p-value) the FDR q-value is (p-value * number of GO terms) / i. Enrichment (N, B, n, b) is defined as follows: N - is the total number of genes; B - is the total number of genes associated with a specific GO term; n - is the number of genes in the top of the user's input list or in the target set when appropriate; b - is the number of genes in the intersection. Enrichment = (b/n) / (B/N). The genes classified in each GO term are also listed.

| **GO term** | **Description** | [**P-value**](http://cbl-gorilla.cs.technion.ac.il/GOrilla/akje8mfg/GOResultsFUNCTION.html#p_value_info) | [**FDR q-value**](http://cbl-gorilla.cs.technion.ac.il/GOrilla/akje8mfg/GOResultsFUNCTION.html#fdr_info) | [**Enrichment (N, B, n, b)**](http://cbl-gorilla.cs.technion.ac.il/GOrilla/akje8mfg/GOResultsFUNCTION.html#enrich_info) | [**Genes**](http://cbl-gorilla.cs.technion.ac.il/GOrilla/akje8mfg/GOResultsFUNCTION.html#genes_info) |
| --- | --- | --- | --- | --- | --- |
| [GO:0048037](http://www.godatabase.org/cgi-bin/amigo/go.cgi?query=GO:0048037&view=details) | cofactor binding | 3.52E-9 | 6.12E-6 | 5.57 (11861,238,161,18) | [[-] Hide genes](javascript:toggle('elements_GO:0048037'))  AT4G08780 - peroxidase 38 AT5G06730 - peroxidase AT5G19880 - peroxidase AT5G05340 - peroxidase 52 AT2G46750 - d-arabinono-1,4-lactone oxidase-like protein AT5G38710 - proline dehydrogenase 2 AT1G26380 - fad-binding and bbe domain-containing protein AT1G34510 - peroxidase 8 AT5G44400 - fad-binding and bbe domain-containing protein AT1G26390 - fad-binding berberine family protein AT4G36430 - peroxidase 49 AT1G26420 - fad-binding and bbe domain-containing protein AT1G26410 - fad-binding and bbe domain-containing protein AT1G12200 - putative flavin monooxygenase. AT4G12280 - copper amine oxidase family protein AT1G14550 - peroxidase 5 AT5G39580 - peroxidase 62 AT1G14540 - peroxidase 4 |
| [GO:0004601](http://www.godatabase.org/cgi-bin/amigo/go.cgi?query=GO:0004601&view=details) | peroxidase activity | 1.19E-7 | 1.03E-4 | 10.87 (11861,61,161,9) | [[-] Hide genes](javascript:toggle('elements_GO:0004601'))  AT4G08780 - peroxidase 38 AT1G34510 - peroxidase 8 AT5G06730 - peroxidase AT4G36430 - peroxidase 49 AT5G19880 - peroxidase AT5G05340 - peroxidase 52 AT1G14550 - peroxidase 5 AT1G14540 - peroxidase 4 AT5G39580 - peroxidase 62 |
| [GO:0016684](http://www.godatabase.org/cgi-bin/amigo/go.cgi?query=GO:0016684&view=details) | oxidoreductase activity, acting on peroxide as acceptor | 1.37E-7 | 7.94E-5 | 10.69 (11861,62,161,9) | [[-] Hide genes](javascript:toggle('elements_GO:0016684'))  AT4G08780 - peroxidase 38 AT1G34510 - peroxidase 8 AT5G06730 - peroxidase AT4G36430 - peroxidase 49 AT5G05340 - peroxidase 52 AT5G19880 - peroxidase AT1G14550 - peroxidase 5 AT1G14540 - peroxidase 4 AT5G39580 - peroxidase 62 |
| [GO:0071949](http://www.godatabase.org/cgi-bin/amigo/go.cgi?query=GO:0071949&view=details) | FAD binding | 2.37E-7 | 1.03E-4 | 15.63 (11861,33,161,7) | [[-] Hide genes](javascript:toggle('elements_GO:0071949'))  AT1G26380 - fad-binding and bbe domain-containing protein AT5G44400 - fad-binding and bbe domain-containing protein AT1G26390 - fad-binding berberine family protein AT1G26420 - fad-binding and bbe domain-containing protein AT1G26410 - fad-binding and bbe domain-containing protein AT2G46750 - d-arabinono-1,4-lactone oxidase-like protein AT5G38710 - proline dehydrogenase 2 |
| [GO:0016209](http://www.godatabase.org/cgi-bin/amigo/go.cgi?query=GO:0016209&view=details) | antioxidant activity | 3.11E-7 | 1.08E-4 | 9.75 (11861,68,161,9) | [[-] Hide genes](javascript:toggle('elements_GO:0016209'))  AT4G08780 - peroxidase 38 AT1G34510 - peroxidase 8 AT5G06730 - peroxidase AT4G36430 - peroxidase 49 AT5G19880 - peroxidase AT5G05340 - peroxidase 52 AT1G14550 - peroxidase 5 AT1G14540 - peroxidase 4 AT5G39580 - peroxidase 62 |
| [GO:0020037](http://www.godatabase.org/cgi-bin/amigo/go.cgi?query=GO:0020037&view=details) | heme binding | 4.01E-7 | 1.16E-4 | 9.47 (11861,70,161,9) | [[-] Hide genes](javascript:toggle('elements_GO:0020037'))  AT4G08780 - peroxidase 38 AT1G34510 - peroxidase 8 AT5G06730 - peroxidase AT4G36430 - peroxidase 49 AT5G19880 - peroxidase AT5G05340 - peroxidase 52 AT1G14550 - peroxidase 5 AT5G39580 - peroxidase 62 AT1G14540 - peroxidase 4 |
| [GO:0046906](http://www.godatabase.org/cgi-bin/amigo/go.cgi?query=GO:0046906&view=details) | tetrapyrrole binding | 4.54E-7 | 1.13E-4 | 9.34 (11861,71,161,9) | [[-] Hide genes](javascript:toggle('elements_GO:0046906'))  AT4G08780 - peroxidase 38 AT1G34510 - peroxidase 8 AT5G06730 - peroxidase AT4G36430 - peroxidase 49 AT5G05340 - peroxidase 52 AT5G19880 - peroxidase AT1G14550 - peroxidase 5 AT5G39580 - peroxidase 62 AT1G14540 - peroxidase 4 |
| [GO:0050660](http://www.godatabase.org/cgi-bin/amigo/go.cgi?query=GO:0050660&view=details) | flavin adenine dinucleotide binding | 1.19E-6 | 2.59E-4 | 9.99 (11861,59,161,8) | [[-] Hide genes](javascript:toggle('elements_GO:0050660'))  AT1G26380 - fad-binding and bbe domain-containing protein AT5G44400 - fad-binding and bbe domain-containing protein AT1G26390 - fad-binding berberine family protein AT1G26420 - fad-binding and bbe domain-containing protein AT1G26410 - fad-binding and bbe domain-containing protein AT1G12200 - putative flavin monooxygenase. AT2G46750 - d-arabinono-1,4-lactone oxidase-like protein AT5G38710 - proline dehydrogenase 2 |
| [GO:0050662](http://www.godatabase.org/cgi-bin/amigo/go.cgi?query=GO:0050662&view=details) | coenzyme binding | 2.97E-4 | 5.73E-2 | 4.71 (11861,125,161,8) | [[-] Hide genes](javascript:toggle('elements_GO:0050662'))  AT1G26380 - fad-binding and bbe domain-containing protein AT5G44400 - fad-binding and bbe domain-containing protein AT1G26390 - fad-binding berberine family protein AT1G26420 - fad-binding and bbe domain-containing protein AT1G26410 - fad-binding and bbe domain-containing protein AT1G12200 - putative flavin monooxygenase. AT2G46750 - d-arabinono-1,4-lactone oxidase-like protein AT5G38710 - proline dehydrogenase 2 |
| [GO:0005388](http://www.godatabase.org/cgi-bin/amigo/go.cgi?query=GO:0005388&view=details) | calcium-transporting ATPase activity | 5.44E-4 | 9.46E-2 | 49.11 (11861,3,161,2) | [[-] Hide genes](javascript:toggle('elements_GO:0005388'))  AT3G22910 - putative calcium-transporting atpase 13 AT3G63380 - putative calcium-transporting atpase 12 |
| [GO:0016491](http://www.godatabase.org/cgi-bin/amigo/go.cgi?query=GO:0016491&view=details) | oxidoreductase activity | 7.52E-4 | 1.19E-1 | 2.57 (11861,430,161,15) | [[-] Hide genes](javascript:toggle('elements_GO:0016491'))  AT4G08780 - peroxidase 38 AT5G06730 - peroxidase AT5G05340 - peroxidase 52 AT5G19880 - peroxidase AT2G46750 - d-arabinono-1,4-lactone oxidase-like protein AT1G66800 - alcohol dehydrogenase-like protein AT5G38710 - proline dehydrogenase 2 AT1G34510 - peroxidase 8 AT4G36430 - peroxidase 49 AT1G12200 - putative flavin monooxygenase. AT4G12280 - copper amine oxidase family protein AT1G14550 - peroxidase 5 AT5G39580 - peroxidase 62 AT5G38900 - thioredoxin superfamily protein AT1G14540 - peroxidase 4 |
