# Supplementary material for: Evidences for a Nutritional Role of Iodine in Plants
Source: Front Plant Sci. 2021 Feb 17;12:616868. doi: 10.3389/fpls.2021.616868 (PMC7925997; doi:10.3389/fpls.2021.616868)
Supplement: Supplementary file 20 [file Table_12.DOCX]

**Table S12.** List of the biological processes and molecular functions affected by protein iodination in leaves (chloroplast, cauline and rosette), based on the GO terms enrichment analysis performed through String platform (https://string-db.org). The list of 42 proteins iodinated in leaves was used as input and the enrichment analysis was performed against the whole genome as statistical background. The GO term ID, GO term description, the number of proteins from the input list classified in each GO term and the number of background proteins associated with a specific GO term are reported, with the false discovery rate (FDR) value

| LEAVES – Biological processes (GO terms enrichment)  *OGC: observed gene count; **BGC: background gene count | | | | | | | | |
| --- | --- | --- | --- | --- | --- | --- | --- | --- |
| #term ID | term description | | OGC * | | BGC** | | FDR | matching proteins in your network (labels) |
| GO:0015979 | photosynthesis | | 31 | | 215 | | 7.82e-53 | AT1G74470,ATPC1,CAB3,FNR1,LHCB2.1,LHCB3,LHCB5,PETA,PETC,PGRL1A,PSAB,PSAE-1,PSAE-2,PSAF,PSAH2,PSB27,PSB29,PSBA,PSBB,PSBC,PSBD,PSBO2,PSBP-1,PSBP-2,PSBQ-2,PSBQA,PSBR,RBCL,RBCS1A,TLP18.3,VAR1 |
| GO:0019684 | photosynthesis, light reaction | | 20 | | 98 | | 8.52e-35 | ATPC1,CAB3,FNR1,LHCB2.1,LHCB3,LHCB5,PETA,PETC,PGRL1A,PSB27,PSB29,PSBA,PSBB,PSBC,PSBD,PSBO2,PSBP-1,PSBR,TLP18.3,VAR1 |
| GO:0006091 | generation of precursor metabolites and energy | | 22 | | 360 | | 4.38e-28 | ATPC1,CAB3,DRT112,FBA2,FNR1,LHCB2.1,LHCB3,LHCB5,PETA,PETC,PGRL1A,PSB27,PSB29,PSBA,PSBB,PSBC,PSBD,PSBO2,PSBP-1,PSBR,TLP18.3,VAR1 |
| GO:0009767 | photosynthetic electron transport chain | | 9 | | 40 | | 3.80e-15 | ATPC1,FNR1,PETA,PETC,PGRL1A,PSBA,PSBB,PSBC,PSBD |
| GO:0018298 | protein-chromophore linkage | | 9 | | 43 | | 5.43e-15 | CAB3,LHCB2.1,LHCB3,LHCB5,PSAB,PSBA,PSBB,PSBC,PSBD |
| GO:0022900 | electron transport chain | | 10 | | 160 | | 5.55e-12 | ATPC1,DRT112,FNR1,PETA,PETC,PGRL1A,PSBA,PSBB,PSBC,PSBD |
| GO:0044237 | cellular metabolic process | | 36 | | 8432 | | 1.45e-11 | AT1G74470,ATPA,ATPC1,CAB3,DRT112,FBA2,FNR1,LHCB2.1,LHCB3,LHCB5,PB,PETA,PETC,PGRL1A,PSAB,PSAE-1,PSAE-2,PSAF,PSAH2,PSB27,PSB29,PSBA,PSBB,PSBC,PSBD,PSBO2,PSBP-1,PSBP-2,PSBQ-2,PSBQA,PSBR,PYK10,RBCL,RBCS1A,TLP18.3,VAR1 |
| GO:0009765 | photosynthesis, light harvesting | | 7 | | 35 | | 1.59e-11 | CAB3,LHCB2.1,LHCB3,LHCB5,PSB27,TLP18.3,VAR1 |
| GO:0010207 | photosystem II assembly | | 6 | | 15 | | 2.21e-11 | LHCB5,PSB27,PSB29,PSBB,PSBO2,PSBR |
| GO:0009772 | photosynthetic electron transport in photosystem II | | 5 | | 8 | | 3.53e-10 | ATPC1,PSBA,PSBB,PSBC,PSBD |
| GO:0055114 | oxidation-reduction process | | 15 | | 1348 | | 2.30e-08 | AT1G74470,ATPC1,DRT112,FNR1,PETA,PETC,PGRL1A,PSAB,PSBA,PSBB,PSBC,PSBD,PSBO2,RBCL,RBCS1A |
| GO:0050896 | response to stimulus | | 25 | | 5064 | | 1.34e-07 | AT5G07020,ATPA,CAB3,DRT112,FBA2,FNR1,LHCB2.1,LHCB3,LHCB5,PB,PETC,PSAE-1,PSAE-2,PSAH2,PSB27,PSB29,PSBA,PSBO2,PSBP-1,PSBR,PYK10,RBCL,RBCS1A,RCA,VAR1 |
| GO:0009416 | response to light stimulus | | 10 | | 585 | | 4.71e-07 | CAB3,LHCB2.1,LHCB3,LHCB5,PETC,PSB27,PSBO2,RBCS1A,RCA,VAR1 |
| GO:0009735 | response to cytokinin | | 7 | | 212 | | 9.89e-07 | AT5G07020,PSAE-1,PSAE-2,PSAH2,PSBR,PYK10,VAR1 |
| GO:0009768 | photosynthesis, light harvesting in photosystem I | | 4 | | 19 | | 9.89e-07 | CAB3,LHCB2.1,LHCB3,LHCB5 |
| GO:0034622 | cellular protein-containing complex assembly | | 8 | | 332 | | 9.89e-07 | LHCB5,PSB27,PSB29,PSBB,PSBO2,PSBR,RBCS1A,VAR1 |
| GO:0010033 | response to organic substance | | 14 | | 1786 | | 4.79e-06 | AT5G07020,CAB3,FBA2,LHCB2.1,LHCB3,PSAE-1,PSAE-2,PSAH2,PSB29,PSBR,PYK10,RBCL,RCA,VAR1 |
| GO:0042221 | response to chemical | | 16 | | 2654 | | 1.59e-05 | AT5G07020,CAB3,DRT112,FBA2,LHCB2.1,LHCB3,PSAE-1,PSAE-2,PSAH2,PSB29,PSBA,PSBR,PYK10,RBCL,RCA,VAR1 |
| GO:0009628 | response to abiotic stimulus | | 13 | | 1699 | | 1.68e-05 | ATPA,CAB3,LHCB2.1,LHCB3,LHCB5,PB,PETC,PSB27,PSBO2,PYK10,RBCS1A,RCA,VAR1 |
| GO:0009725 | response to hormone | | 12 | | 1502 | | 3.02e-05 | AT5G07020,FBA2,LHCB2.1,LHCB3,PSAE-1,PSAE-2,PSAH2,PSBR,PYK10,RBCL,RCA,VAR1 |
| GO:0010218 | response to far red light | | 4 | | 57 | | 3.50e-05 | CAB3,LHCB2.1,LHCB5,RBCS1A |
| GO:0010206 | photosystem II repair | | 3 | | 15 | | 3.90e-05 | PSB27,TLP18.3,VAR1 |
| GO:0009642 | response to light intensity | | 5 | | 142 | | 4.20e-05 | LHCB2.1,LHCB3,PSB27,PSBO2,VAR1 |
| GO:1901564 | organonitrogen compound metabolic process | | 19 | | 4116 | | 4.20e-05 | AT1G74470,ATPA,ATPC1,CAB3,FBA2,LHCB2.1,LHCB3,LHCB5,PB,PSAB,PSB27,PSB29,PSBA,PSBB,PSBC,PSBD,PYK10,TLP18.3,VAR1 |
| GO:0010114 | response to red light | | 4 | | 64 | | 4.74e-05 | CAB3,LHCB2.1,LHCB5,RBCS1A |
| GO:0009644 | response to high light intensity | | 4 | | 76 | | 8.81e-05 | LHCB2.1,LHCB3,PSBO2,VAR1 |
| GO:0009637 | response to blue light | | 4 | | 80 | | 0.00010 | CAB3,LHCB2.1,LHCB5,RBCS1A |
| GO:0006754 | ATP biosynthetic process | | 4 | | 89 | | 0.00015 | ATPA,ATPC1,FBA2,PB |
| GO:0009409 | response to cold | | 6 | | 347 | | 0.00018 | ATPA,LHCB2.1,PB,PYK10,RBCS1A,RCA |
| GO:0015986 | ATP synthesis coupled proton transport | | 3 | | 31 | | 0.00020 | ATPA,ATPC1,PB |
| GO:0009168 | purine ribonucleoside monophosphate biosynthetic process | | 4 | | 110 | | 0.00027 | ATPA,ATPC1,FBA2,PB |
| GO:0009769 | photosynthesis, light harvesting in photosystem II | | 2 | | 4 | | 0.00031 | LHCB2.1,LHCB3 |
| GO:0010196 | nonphotochemical quenching | | 2 | | 6 | | 0.00052 | LHCB5,PETC |
| GO:1903426 | regulation of reactive oxygen species biosynthetic process | | 2 | | 7 | | 0.00059 | LHCB2.1,PSB29 |
| GO:0042742 | defense response to bacterium | | 5 | | 315 | | 0.00080 | ATPA,FNR1,PETC,PSBP-1,RCA |
| GO:0010119 | regulation of stomatal movement | | 3 | | 70 | | 0.0012 | LHCB2.1,LHCB3,PSB29 |
| GO:0009635 | response to herbicide | | 2 | | 13 | | 0.0014 | LHCB3,PSBA |
| GO:0010205 | photoinhibition | | 2 | | 14 | | 0.0015 | PSBO2,VAR1 |
| GO:0090662 | ATP hydrolysis coupled transmembrane transport | | 3 | | 77 | | 0.0015 | ATPA,ATPC1,PB |
| GO:0099131 | ATP hydrolysis coupled ion transmembrane transport | | 3 | | 77 | | 0.0015 | ATPA,ATPC1,PB |
| GO:0099132 | ATP hydrolysis coupled cation transmembrane transport | | 3 | | 78 | | 0.0015 | ATPA,ATPC1,PB |
| GO:0019253 | reductive pentose-phosphate cycle | | 2 | | 20 | | 0.0026 | RBCL,RBCS1A |
| GO:0044267 | cellular protein metabolic process | | 12 | | 2826 | | 0.0039 | CAB3,LHCB2.1,LHCB3,LHCB5,PSAB,PSB27,PSBA,PSBB,PSBC,PSBD,TLP18.3,VAR1 |
| GO:0051707 | response to other organism | | 7 | | 1079 | | 0.0055 | ATPA,FNR1,PB,PETC,PSBP-1,PYK10,RCA |
| GO:0098542 | defense response to other organism | | 6 | | 847 | | 0.0078 | ATPA,FNR1,PB,PETC,PSBP-1,RCA |
| GO:0009853 | photorespiration | | 2 | | 49 | | 0.0115 | RBCL,RBCS1A |
| GO:0015994 | chlorophyll metabolic process | | 2 | | 56 | | 0.0145 | AT1G74470,PSB29 |
| GO:0006950 | response to stress | | 11 | | 2932 | | 0.0154 | ATPA,FNR1,LHCB2.1,LHCB3,PB,PETC,PSBA,PSBP-1,PYK10,RBCS1A,RCA |
| GO:1901135 | carbohydrate derivative metabolic process | | 5 | | 701 | | 0.0170 | ATPA,ATPC1,FBA2,PB,PYK10 |
| GO:0044270 | cellular nitrogen compound catabolic process | | 3 | | 214 | | 0.0174 | FBA2,PSB29,PYK10 |
| GO:0046700 | heterocycle catabolic process | | 3 | | 214 | | 0.0174 | FBA2,PSB29,PYK10 |
| GO:1901700 | response to oxygen-containing compound | | 7 | | 1398 | | 0.0191 | CAB3,FBA2,LHCB2.1,LHCB3,PSB29,RBCL,RCA |
| GO:0019439 | aromatic compound catabolic process | | 3 | | 238 | | 0.0214 | FBA2,PSB29,PYK10 |
| GO:0016043 | cellular component organization | | 9 | | 2271 | | 0.0237 | LHCB5,PSB27,PSB29,PSBB,PSBO2,PSBR,PYK10,RBCS1A,VAR1 |
| GO:0051704 | multi-organism process | | 7 | | 1475 | | 0.0237 | ATPA,FNR1,PB,PETC,PSBP-1,PYK10,RCA |
| GO:1901361 | organic cyclic compound catabolic process | | 3 | | 249 | | 0.0237 | FBA2,PSB29,PYK10 |
| GO:0009737 | response to abscisic acid | | 4 | | 511 | | 0.0268 | FBA2,LHCB2.1,LHCB3,RBCL |
| GO:0071704 | organic substance metabolic process | | 21 | | 8632 | | 0.0319 | AT1G74470,ATPA,ATPC1,CAB3,FBA2,LHCB2.1,LHCB3,LHCB5,PB,PSAB,PSB27,PSB29,PSBA,PSBB,PSBC,PSBD,PYK10,RBCL,RBCS1A,TLP18.3,VAR1 |
| GO:0016051 | carbohydrate biosynthetic process | | 3 | | 295 | | 0.0351 | FBA2,RBCL,RBCS1A |
| GO:0017144 | drug metabolic process | | 4 | | 626 | | 0.0498 | ATPA,ATPC1,FBA2,PB |
|  | | | | | | | | |
| LEAVES – Molecular functions (GO terms enrichment)  *OGC: observed gene count; **BGC: background gene count | | | | | | | | |
| #term ID | term description | OGC * | | BGC** | | FDR | | matching proteins in your network (labels) |
| GO:0016168 | chlorophyll binding | 9 | | 30 | | 4.35e-16 | | CAB3,LHCB2.1,LHCB3,LHCB5,PSAB,PSBA,PSBB,PSBC,PSBD |
| GO:0019904 | protein domain specific binding | 10 | | 71 | | 2.22e-15 | | DRT112,FNR1,LHCB2.1,LHCB3,LHCB5,PGRL1A,PSAE-1,PSAE-2,PSAF,PSBP-1 |
| GO:0046906 | tetrapyrrole binding | 11 | | 299 | | 3.86e-11 | | CAB3,LHCB2.1,LHCB3,LHCB5,MAPR2,PETA,PSAB,PSBA,PSBB,PSBC,PSBD |
| GO:0009055 | electron transfer activity | 8 | | 105 | | 1.95e-10 | | DRT112,FNR1,PETA,PETC,PSBA,PSBB,PSBC,PSBD |
| GO:0045156 | electron transporter, transferring electrons within the cyclic electron transport pathway of photosynthesis activity | 5 | | 9 | | 3.01e-10 | | FNR1,PSBA,PSBB,PSBC,PSBD |
| GO:0046872 | metal ion binding | 22 | | 2940 | | 5.68e-10 | | ATPA,CAB3,DRT112,LHCB2.1,LHCB3,LHCB5,ORF110A,PB,PETA,PETC,PSAB,PSBA,PSBC,PSBD,PSBP-1,PSBP-2,PSBQ-2,PSBQA,PYK10,RBCL,RBCS1A,VAR1 |
| GO:0016491 | oxidoreductase activity | 14 | | 1201 | | 2.46e-08 | | AT1G74470,DRT112,FNR1,PETA,PETC,PGRL1A,PSAB,PSBA,PSBB,PSBC,PSBD,PSBO2,RBCL,RBCS1A |
| GO:0048037 | cofactor binding | 12 | | 860 | | 5.80e-08 | | CAB3,LHCB2.1,LHCB3,LHCB5,MAPR2,PETA,PETC,PSAB,PSBA,PSBB,PSBC,PSBD |
| GO:0005488 | binding | 31 | | 8611 | | 2.45e-07 | | ATPA,CAB3,DRT112,FNR1,LHCB2.1,LHCB3,LHCB5,MAPR2,ORF110A,PB,PETA,PETC,PGRL1A,PSAB,PSAE-1,PSAE-2,PSAF,PSBA,PSBB,PSBC,PSBD,PSBO2,PSBP-1,PSBP-2,PSBQ-2,PSBQA,PYK10,RBCL,RBCS1A,RCA,VAR1 |
| GO:0043167 | ion binding | 24 | | 5070 | | 2.97e-07 | | ATPA,CAB3,DRT112,LHCB2.1,LHCB3,LHCB5,ORF110A,PB,PETA,PETC,PSAB,PSBA,PSBB,PSBC,PSBD,PSBP-1,PSBP-2,PSBQ-2,PSBQA,PYK10,RBCL,RBCS1A,RCA,VAR1 |
| GO:0031409 | pigment binding | 4 | | 19 | | 3.96e-07 | | CAB3,LHCB2.1,LHCB3,LHCB5 |
| GO:0008266 | poly(U) RNA binding | 3 | | 16 | | 2.83e-05 | | FNR1,PSBO2,PSBP-1 |
| GO:0046933 | proton-transporting ATP synthase activity, rotational mechanism | 3 | | 19 | | 3.88e-05 | | ATPA,ATPC1,PB |
| GO:0046028 | electron transporter, transferring electrons from cytochrome b6/f complex of photosystem II activity | 2 | | 2 | | 9.92e-05 | | DRT112,PETC |
| GO:0044769 | ATPase activity, coupled to transmembrane movement of ions, rotational mechanism | 3 | | 37 | | 0.00022 | | ATPA,ATPC1,PB |
| GO:0016984 | ribulose-bisphosphate carboxylase activity | 2 | | 5 | | 0.00031 | | RBCL,RBCS1A |
| GO:0016730 | oxidoreductase activity, acting on iron-sulfur proteins as donors | 2 | | 8 | | 0.00059 | | FNR1,PGRL1A |
| GO:0043168 | anion binding | 13 | | 2629 | | 0.00059 | | ATPA,CAB3,LHCB2.1,LHCB3,LHCB5,PB,PSAB,PSBA,PSBB,PSBC,PSBD,RCA,VAR1 |
| GO:0005515 | protein binding | 11 | | 1988 | | 0.00081 | | DRT112,FNR1,LHCB2.1,LHCB3,LHCB5,PGRL1A,PSAE-1,PSAE-2,PSAF,PSBP-1,PYK10 |
| GO:0019829 | cation-transporting ATPase activity | 3 | | 74 | | 0.0011 | | ATPA,ATPC1,PB |
| GO:0005509 | calcium ion binding | 4 | | 226 | | 0.0019 | | PSBP-1,PSBP-2,PSBQ-2,PSBQA |
| GO:0016830 | carbon-carbon lyase activity | 3 | | 96 | | 0.0020 | | FBA2,RBCL,RBCS1A |
| GO:0003824 | catalytic activity | 21 | | 7239 | | 0.0039 | | AT1G74470,ATPA,ATPC1,DRT112,FBA2,FNR1,PB,PETA,PETC,PGRL1A,PSAB,PSBA,PSBB,PSBC,PSBD,PSBO2,PYK10,RBCL,RBCS1A,TLP18.3,VAR1 |
| GO:0097159 | organic cyclic compound binding | 18 | | 5841 | | 0.0055 | | ATPA,CAB3,FNR1,LHCB2.1,LHCB3,LHCB5,MAPR2,PB,PETA,PSAB,PSBA,PSBB,PSBC,PSBD,PSBO2,PSBP-1,RCA,VAR1 |
| GO:1901363 | heterocyclic compound binding | 18 | | 5835 | | 0.0055 | | ATPA,CAB3,FNR1,LHCB2.1,LHCB3,LHCB5,MAPR2,PB,PETA,PSAB,PSBA,PSBB,PSBC,PSBD,PSBO2,PSBP-1,RCA,VAR1 |
| GO:0005507 | copper ion binding | 3 | | 157 | | 0.0071 | | DRT112,PYK10,RBCS1A |
| GO:0046914 | transition metal ion binding | 6 | | 933 | | 0.0101 | | ATPA,DRT112,PB,PETA,PYK10,RBCS1A |
| GO:0042623 | ATPase activity, coupled | 4 | | 391 | | 0.0105 | | ATPA,ATPC1,PB,VAR1 |
| GO:0043492 | ATPase activity, coupled to movement of substances | 3 | | 199 | | 0.0118 | | ATPA,ATPC1,PB |
| GO:0015405 | P-P-bond-hydrolysis-driven transmembrane transporter activity | 3 | | 207 | | 0.0127 | | ATPA,ATPC1,PB |
| GO:0016887 | ATPase activity | 4 | | 498 | | 0.0202 | | ATPA,ATPC1,PB,VAR1 |
| GO:0000287 | magnesium ion binding | 2 | | 115 | | 0.0374 | | ORF110A,RBCL |
