# Supplementary material for: Evidences for a Nutritional Role of Iodine in Plants
Source: Front Plant Sci. 2021 Feb 17;12:616868. doi: 10.3389/fpls.2021.616868 (PMC7925997; doi:10.3389/fpls.2021.616868)
Supplement: Supplementary file 22 [file Table_14.DOCX]

**Table S14.** List of the biological processes and molecular functions affected by protein iodination in roots, based on the GO terms enrichment analysis performed through String platform (https://string-db.org). The list of 40 proteins iodinated in leaves was used as input and the enrichment analysis was performed against the whole genome as statistical background. The GO term ID, GO term description, the number of proteins from the input list classified in each GO term and the number of background proteins associated with a specific GO term are reported, with the false discovery rate (FDR) value

| ROOTS – Biological processes (GO terms enrichment)  *OGC: observed gene count; **BGC: background gene count | | | | | |
| --- | --- | --- | --- | --- | --- |
| #term ID | term description | OGC * | BGC** | FDR | matching proteins in your network (labels) |
| GO:0017144 | drug metabolic process | 11 | 626 | 3.24e-07 | AT2G37130,AT2G43610,AT3G01190,AT4G30170,AT5G08680,AT5G17820,FUM2,GAPC1,P1,PGK,TPI |
| GO:0006950 | response to stress | 20 | 2932 | 3.24e-07 | ACT7,AT2G37130,AT2G43610,AT3G01190,AT4G16260,AT4G20830,AT4G30170,AT5G17820,AT5G20080,AT5G44380,ATBFRUCT1,BIP2,FUM2,GAPC1,HSP70,JAL34,P1,PCAP1,PGK,TPI |
| GO:0006979 | response to oxidative stress | 9 | 389 | 7.87e-07 | AT2G37130,AT3G01190,AT4G20830,AT4G30170,AT5G17820,AT5G44380,GAPC1,HSP70,P1 |
| GO:0009636 | response to toxic substance | 8 | 330 | 3.30e-06 | AT2G37130,AT3G01190,AT4G30170,AT5G17820,GAPC1,HSP70,P1,PLAT2 |
| GO:0050896 | response to stimulus | 23 | 5064 | 4.53e-06 | ACT7,AGP31,AT2G37130,AT2G43610,AT3G01190,AT4G16260,AT4G20830,AT4G30170,AT5G08680,AT5G17820,AT5G20080,AT5G44380,ATBFRUCT1,BIP2,FUM2,GAPC1,HSP70,JAL34,P1,PCAP1,PGK,PLAT2,TPI |
| GO:0016999 | antibiotic metabolic process | 6 | 171 | 1.75e-05 | AT2G37130,AT3G01190,AT4G30170,AT5G17820,FUM2,P1 |
| GO:0042743 | hydrogen peroxide metabolic process | 5 | 95 | 1.75e-05 | AT2G37130,AT3G01190,AT4G30170,AT5G17820,P1 |
| GO:0042744 | hydrogen peroxide catabolic process | 5 | 88 | 1.75e-05 | AT2G37130,AT3G01190,AT4G30170,AT5G17820,P1 |
| GO:0098869 | cellular oxidant detoxification | 6 | 179 | 1.75e-05 | AT2G37130,AT3G01190,AT4G30170,AT5G17820,P1,PLAT2 |
| GO:0051186 | cofactor metabolic process | 8 | 531 | 3.29e-05 | AT2G37130,AT3G01190,AT4G30170,AT5G17820,GAPC1,P1,PGK,TPI |
| GO:0042737 | drug catabolic process | 6 | 226 | 3.41e-05 | AT2G37130,AT2G43610,AT3G01190,AT4G30170,AT5G17820,P1 |
| GO:0009664 | plant-type cell wall organization | 5 | 139 | 5.80e-05 | AT2G37130,AT3G01190,AT5G17820,P1,XTH24 |
| GO:0042221 | response to chemical | 15 | 2654 | 6.25e-05 | ACT7,AGP31,AT2G37130,AT3G01190,AT4G30170,AT5G08680,AT5G17820,BIP2,GAPC1,HSP70,P1,PCAP1,PGK,PLAT2,TPI |
| GO:0055114 | oxidation-reduction process | 11 | 1348 | 6.39e-05 | AT2G37130,AT3G01190,AT4G20830,AT4G30170,AT5G17820,AT5G20080,AT5G44380,FUM2,GAPC1,P1,PLAT2 |
| GO:0006754 | ATP biosynthetic process | 4 | 89 | 0.00024 | AT5G08680,GAPC1,PGK,TPI |
| GO:0009168 | purine ribonucleoside monophosphate biosynthetic process | 4 | 110 | 0.00044 | AT5G08680,GAPC1,PGK,TPI |
| GO:0009628 | response to abiotic stimulus | 11 | 1699 | 0.00044 | ACT7,AT4G16260,AT5G20080,ATBFRUCT1,FUM2,GAPC1,HSP70,JAL34,PCAP1,PGK,TPI |
| GO:0005975 | carbohydrate metabolic process | 8 | 856 | 0.00049 | AT2G43610,AT4G16260,ATBFRUCT1,BGAL5,GAPC1,PGK,TPI,XTH24 |
| GO:0010038 | response to metal ion | 6 | 414 | 0.00049 | AT5G08680,BIP2,GAPC1,HSP70,PCAP1,TPI |
| GO:0046034 | ATP metabolic process | 4 | 125 | 0.00057 | AT5G08680,GAPC1,PGK,TPI |
| GO:0071554 | cell wall organization or biogenesis | 7 | 639 | 0.00057 | AT2G37130,AT2G43610,AT3G01190,AT4G19410,AT5G17820,P1,XTH24 |
| GO:0046686 | response to cadmium ion | 5 | 286 | 0.00079 | AT5G08680,BIP2,GAPC1,HSP70,TPI |
| GO:0009167 | purine ribonucleoside monophosphate metabolic process | 4 | 149 | 0.00084 | AT5G08680,GAPC1,PGK,TPI |
| GO:0009651 | response to salt stress | 6 | 492 | 0.00085 | AT4G16260,AT5G20080,FUM2,GAPC1,PCAP1,TPI |
| GO:0006096 | glycolytic process | 3 | 57 | 0.00094 | GAPC1,PGK,TPI |
| GO:0006757 | ATP generation from ADP | 3 | 57 | 0.00094 | GAPC1,PGK,TPI |
| GO:0009266 | response to temperature stimulus | 6 | 505 | 0.00094 | FUM2,GAPC1,HSP70,JAL34,PCAP1,PGK |
| GO:0042866 | pyruvate biosynthetic process | 3 | 57 | 0.00094 | GAPC1,PGK,TPI |
| GO:0071555 | cell wall organization | 6 | 503 | 0.00094 | AT2G37130,AT3G01190,AT4G19410,AT5G17820,P1,XTH24 |
| GO:0009166 | nucleotide catabolic process | 3 | 61 | 0.00097 | GAPC1,PGK,TPI |
| GO:0044248 | cellular catabolic process | 9 | 1345 | 0.00097 | AT2G37130,AT2G43610,AT3G01190,AT4G30170,AT5G17820,GAPC1,P1,PGK,TPI |
| GO:0019359 | nicotinamide nucleotide biosynthetic process | 3 | 67 | 0.0011 | GAPC1,PGK,TPI |
| GO:0006090 | pyruvate metabolic process | 3 | 74 | 0.0014 | GAPC1,PGK,TPI |
| GO:0006094 | gluconeogenesis | 2 | 18 | 0.0024 | GAPC1,TPI |
| GO:0032272 | negative regulation of protein polymerization | 2 | 18 | 0.0024 | PCAP1,PRF1 |
| GO:0046496 | nicotinamide nucleotide metabolic process | 3 | 96 | 0.0024 | GAPC1,PGK,TPI |
| GO:0051494 | negative regulation of cytoskeleton organization | 2 | 19 | 0.0025 | PCAP1,PRF1 |
| GO:1902904 | negative regulation of supramolecular fiber organization | 2 | 19 | 0.0025 | PCAP1,PRF1 |
| GO:0016052 | carbohydrate catabolic process | 4 | 244 | 0.0026 | AT2G43610,GAPC1,PGK,TPI |
| GO:0009743 | response to carbohydrate | 3 | 123 | 0.0044 | GAPC1,PCAP1,PGK |
| GO:0051707 | response to other organism | 7 | 1079 | 0.0047 | AT2G37130,AT4G16260,AT4G20830,ATBFRUCT1,HSP70,PCAP1,PGK |
| GO:0002237 | response to molecule of bacterial origin | 2 | 33 | 0.0061 | PCAP1,PGK |
| GO:0008152 | metabolic process | 24 | 9671 | 0.0063 | AT2G37130,AT2G43610,AT3G01190,AT3G19390,AT4G16260,AT4G20830,AT4G26220,AT4G30170,AT5G08680,AT5G17820,AT5G20080,AT5G44380,ATBFRUCT1,BGAL5,BIP2,FUM2,GAPC1,HSP70,P1,PCAP1,PGK,PLAT2,TPI,XTH24 |
| GO:0006006 | glucose metabolic process | 2 | 38 | 0.0076 | GAPC1,TPI |
| GO:0006091 | generation of precursor metabolites and energy | 4 | 360 | 0.0091 | FUM2,GAPC1,PGK,TPI |
| GO:0070887 | cellular response to chemical stimulus | 7 | 1245 | 0.0094 | AT2G37130,AT3G01190,AT4G30170,AT5G17820,P1,PCAP1,PLAT2 |
| GO:0009408 | response to heat | 3 | 184 | 0.0114 | GAPC1,HSP70,PGK |
| GO:0007010 | cytoskeleton organization | 3 | 192 | 0.0126 | ACT7,PCAP1,PRF1 |
| GO:1901135 | carbohydrate derivative metabolic process | 5 | 701 | 0.0147 | AT2G43610,AT5G08680,GAPC1,PGK,TPI |
| GO:0009808 | lignin metabolic process | 2 | 59 | 0.0151 | AT4G26220,P1 |
| GO:0009735 | response to cytokinin | 3 | 212 | 0.0158 | BIP2,PCAP1,TPI |
| GO:0042542 | response to hydrogen peroxide | 2 | 63 | 0.0164 | GAPC1,HSP70 |
| GO:0016043 | cellular component organization | 9 | 2271 | 0.0189 | ACT7,AT2G37130,AT3G01190,AT4G19410,AT5G17820,P1,PCAP1,PRF1,XTH24 |
| GO:0050832 | defense response to fungus | 4 | 478 | 0.0203 | AT2G37130,AT4G16260,AT4G20830,ATBFRUCT1 |
| GO:0098542 | defense response to other organism | 5 | 847 | 0.0283 | AT2G37130,AT4G16260,AT4G20830,ATBFRUCT1,PCAP1 |
| GO:0006952 | defense response | 6 | 1277 | 0.0365 | AT2G37130,AT2G43610,AT4G16260,AT4G20830,ATBFRUCT1,PCAP1 |
| GO:0097435 | supramolecular fiber organization | 2 | 106 | 0.0388 | PCAP1,PRF1 |
| GO:0010033 | response to organic substance | 7 | 1786 | 0.0485 | ACT7,AGP31,BIP2,GAPC1,PCAP1,PGK,TPI |
| GO:0009409 | response to cold | 3 | 347 | 0.0494 | FUM2,JAL34,PCAP1 |
|  | | | | | |

| ROOTS – Molecular functions (GO terms enrichment)  *OGC: observed gene count; **BGC: background gene count | | | | | |
| --- | --- | --- | --- | --- | --- |
| #term ID | term description | OGC * | BGC** | FDR | matching proteins in your network (labels) |
| GO:0005507 | copper ion binding | 7 | 157 | 5.13e-07 | AT5G08680,AT5G20080,GAPC1,JAL31,JAL34,PCAP1,TPI |
| GO:0004601 | peroxidase activity | 6 | 128 | 2.75e-06 | AT2G37130,AT3G01190,AT4G30170,AT5G17820,P1,PLAT2 |
| GO:0020037 | heme binding | 7 | 267 | 4.37e-06 | AT2G37130,AT3G01190,AT4G30170,AT5G17820,MAPR3,P1,PLAT2 |
| GO:0048037 | cofactor binding | 10 | 860 | 7.22e-06 | AT2G37130,AT3G01190,AT4G20830,AT4G30170,AT5G17820,AT5G44380,GAPC1,MAPR3,P1,PLAT2 |
| GO:0016491 | oxidoreductase activity | 10 | 1201 | 0.00012 | AT2G37130,AT3G01190,AT4G20830,AT4G30170,AT5G17820,AT5G20080,AT5G44380,GAPC1,P1,PLAT2 |
| GO:0043167 | ion binding | 19 | 5070 | 0.00043 | ACT7,AT2G37130,AT3G01190,AT4G20830,AT4G26220,AT4G30170,AT5G08680,AT5G17820,AT5G20080,AT5G44380,BIP2,GAPC1,HSP70,JAL31,JAL34,P1,PCAP1,PGK,TPI |
| GO:0004553 | hydrolase activity, hydrolyzing O-glycosyl compounds | 5 | 258 | 0.00053 | AT2G43610,AT4G16260,ATBFRUCT1,BGAL5,XTH24 |
| GO:0005488 | binding | 25 | 8611 | 0.00063 | ACT7,AT1G78850,AT2G37130,AT2G43610,AT3G01190,AT4G16260,AT4G20830,AT4G26220,AT4G30170,AT5G08680,AT5G17820,AT5G20080,AT5G44380,BIP2,GAPC1,HSP70,JAL31,JAL34,MAPR3,P1,PCAP1,PGK,PLAT2,PRF1,TPI |
| GO:0003824 | catalytic activity | 22 | 7239 | 0.0013 | AT2G37130,AT2G43610,AT3G01190,AT3G19390,AT4G16260,AT4G19410,AT4G20830,AT4G26220,AT4G30170,AT5G08680,AT5G17820,AT5G20080,AT5G44380,ATBFRUCT1,BGAL5,FUM2,GAPC1,P1,PGK,PLAT2,TPI,XTH24 |
| GO:0046872 | metal ion binding | 13 | 2940 | 0.0018 | AT2G37130,AT3G01190,AT4G26220,AT4G30170,AT5G08680,AT5G17820,AT5G20080,GAPC1,JAL31,JAL34,P1,PCAP1,TPI |
| GO:0030246 | carbohydrate binding | 4 | 306 | 0.0085 | AT1G78850,AT4G16260,JAL31,JAL34 |
